# Comparative Three Dimensional Evaluation of Skeletal and Dento‐Alveolar Effects Between Tooth‐Borne and Bone‐Anchored Maxillary Expansion for Growing Patients—A Systematic Review and Meta‐Analysis

**DOI:** 10.1111/ocr.70029

**Published:** 2025-09-27

**Authors:** Augustine Ka Chun Yung, Ho Hin Chan, Junqi Liu, Kuo Feng Hung, Zhongyuan Tang, Zhiyi Shan

**Affiliations:** ^1^ Division of Paediatric Dentistry and Orthodontics, Faculty of Dentistry The University of Hong Kong Hong Kong China; ^2^ Division of Applied Oral Sciences & Community Dental Care, Faculty of Dentistry The University of Hong Kong Hong Kong China

**Keywords:** maxillary expansion, meta‐analysis, skeletal anchorage, systematic review, three‐dimensional analyses

## Abstract

Transverse maxillary deficiency, a common skeletal discrepancy among children and adolescents globally, can be treated using either bone‐anchored (BA) and tooth‐borne (TB) maxillary expanders. This systematic review and meta‐analysis evaluated the three‐dimensional (3D) difference in the skeletal and dentoalveolar effects of both expander types in growing patients. A systematic literature search was performed across four electronic databases to identify randomised clinical trials that evaluated the skeletal and dentoalveolar effects of BA and TB maxillary expanders using 3D radiographic images with comparisons made immediately after expansion, within 6 months, and beyond 6 months. Following risk of bias assessment, dimensional changes in sutural expansion (SE), nasal width (NW), maxillary width (MW), as well as tooth inclination (TI) and buccal bone thickness (BBT) of posterior tooth regions were extracted and pooled for further meta‐analyses. The analyses were performed based on evaluation periods to determine immediate, short‐term, and long‐term effects. Among 1432 records that have been retrieved, ten studies followed our predetermined eligibility criteria, thus included in this systematic review for qualitative synthesis. Nine of them were able to undergo quantitative synthesis. Regarding the immediate effect, BA expanders presented significantly higher SE, NW, and MW changes than TB expanders, while showing no significant difference in TI and BBT at the molar region. During the first 6 months after expansion (short‐term), evidence has shown that the skeletal and dentoalveolar effects were more favourable to BA expanders in terms of greater nasal width changes and less TI for premolars and molars. However, there were particularly insufficient studies that investigated the long‐term skeletal and dentoalveolar effect differences, though there might be greater nasal width changes after 6 months. Current evidence suggests that hybrid/BA expanders produce significantly greater skeletal effect than tooth‐borne expanders in the immediate and short term. These effects include increased sutural opening and nasal width, alongside less premolar buccal tipping and greater buccal bone thickness at the premolar and molar region less than 6 months after expansion. Over time, the differences between groups appear to diminish time, indicating potential instability in the long‐term outcomes. Due to the high heterogeneity among studies and low quality of evidence reported, these findings should be interpreted with caution. Further studies with more standardised ways would be needed, particularly for long‐term changes.

**Registration:**

PROSPERO (CRD42023399235).

## 1 Introduction

Transverse maxillary deficiency is one of the common skeletal discrepancies seen among children and adolescents, often treated with maxillary expansion [1]. One conventional design of maxillary expanders is the tooth‐borne (TB) type, that is, placing an expansion screw in the mid palate by anchoring to the maxilla via posterior teeth. The treatment effect includes correcting posterior crossbite, increasing the maxillary and nasal width [2, 3], downward rotation of mandible [4] and elevating facial heights [4]. However, TB expanders can cause adverse effects such as buccal tipping of posterior teeth [5], reduced buccal bone thickness, marginal bone loss, occurrences of bone dehiscence [6], and gingival recession [7].

With the introduction of skeletal anchorage, bone‐anchored (BA) or mini‐screw assisted maxillary expanders have been explored as alternatives [8]. BA expanders, encompassing both bone‐borne and tooth‐bone‐borne (hybrid) expanders, rely solely or partly on skeletal anchorage, in contrast to TB expanders that utilise teeth as anchors. Despite this, the comparative effects of BA maxillary expansion with TB expanders remain a subject of debate. Some studies have advocated that BA maxillary expanders have more significant skeletal expansion of the maxilla and reduced adverse dento‐alveolar effects on the anchored and posterior teeth [9, 10, 11, 12, 13, 14] compared to TB expanders in adolescents [15, 16]. Sutural re‐opening was reported in late adolescents and young adults [17, 18, 19]. However, some other studies revealed that they have similar treatment effects [20, 21, 22], with similar skeletal and dental treatment results. An increasing number of studies used 3D radiographic techniques—cone‐beam computed tomography (CBCT) to overcome the limitations of other assessment approaches, which enable precise 3D measurement of skeletal and dentoalveolar effects of maxillary expansion [23], with great clinical significance [24, 25].

To date, four systematic reviews have analysed previous evidence on the comparative treatment effects of BA and TB maxillary expansion. A systematic review in 2019 [26] analysed the randomised controlled trials (RCTs) and revealed that both types of expanders resulted in the same amount of maxillary expansion and dental tipping [26]. However, this review could not perform a meta‐analysis due to the high heterogeneity of the included studies, which involved both children and adults without standardising the radiographic assessment approaches. Another systematic review in 2019 by Krusi et al. [27] reported that the BA expanders might offer benefits over TB expanders in terms of the increased suture expansion, reduced tooth tipping, and lower nasal airway resistance. However, this review is limited in the number of existing studies at that time. Only six RCTs were included, and just three assessed the skeletal effects, making quantitative data synthesis unfeasible. In the systematic review published in 2021, Coloccia et al. [28] included fourteen studies without meta‐analysis. Ten of these were observational studies, which also downgrades the evidence that they provided. Lastly, although Bi et al. [29] conducted a systematic review and meta‐analysis focusing only on RCTs, the included studies have significant methodological heterogeneity, combining data from both 2D and 3D radiographic assessments and various age groups. In recent years, more RCTs have been published that investigated the difference in skeletal and alveolar effects between BA and TB using 3D radiographic images. Therefore, it is imperative to conduct an updated systematic review and meta‐analysis to pool current evidence with 3D radiographic evaluations and homogeneous selection criteria.

## 2 Material and Methods

### 2.1 Protocol and Registration

This systematic review was conducted following the guidelines of the Cochrane Handbook for Systematic Reviews of interventions [30] and was reported using the updated Preferred Reporting Items for Systematic Reviews and Meta‐Analyses (PRISMA) statement [31, 32]. The protocol of this review was made a priori, registered in the PROSPERO database (CRD42023399235).

### 2.2 Research Questions

What are the skeletal and dentoalveolar effects between bone‐anchored (BA) maxillary expansion and tooth‐borne (TB) maxillary expansion in growing patients in the immediate, short, and long terms?

### 2.3 Eligibility Criteria

The eligibility criteria were formulated following the Participants‐Interventions‐Comparisons‐Outcome‐Study design (PICOS) approach. Inclusion and exclusion criteria are listed in Table 1.

**TABLE 1:** The inclusion and exclusion criteria for selection of studies.

Component	Inclusion criteria	Exclusion criteria
Participants	Growing patients with transverse maxillary deficiency No limitation on gender and ethnicity	Patients with cleft lip and palate, craniofacial syndromes, temporomandibular joint disorders, etc. Patients with compromised medical health or systemic illness that require taking medications that might influence the growth or bone metabolism
Intervention	Bone‐anchored maxillary expanders (BA) including the bone‐borne and tooth‐bone‐borne (hybrid) designs	Surgical treatments, including corticectomy, orthognathic surgery, surgically assisted maxillary expansion, etc. Additional orthopaedic appliance during the study period apart from expansion, such as facemask or headgear therapy
Comparison	Tooth‐borne maxillary expander (TB)	Self control Removal maxillary expander Tissue‐borne or tooth‐tissue‐borne maxillary expander
Outcome	Immediate, short‐term, and long‐term effects on skeletal and dentoalveolar bone assessed by cone‐beam computer tomography (CBCT)	Two‐dimensional radiographic evaluations Intraoral scanning model, dental cast, or magnetic resonance imaging
Study design	Randomised controlled trials (RCTs)	Case reports or case series Summary articles, communication, opinion, and letters to the editor Review articles Non‐randomised clinical studies Animal or in vitro studies

### 2.4 Sources of Information, Search Strategy and Study Selection

Four electronic databases were systematically searched for this review: MEDLINE (via PubMed, 1946‐), Embase (1947‐), Cochrane Central Register of Controlled Trials (CENTRAL), and Web of Science were initially searched on 16th April 2023 and updated on 27th May 2024 without language or date restrictions. The search consisted of a combination of three keywords or their synonyms: (1) palatal/maxillary/transverse expansion; (2) computed/digital volume/volumetric tomography; (3) adolescence/children/teen/grow. Full details of the search strategies are presented in Table S1. In addition, supplementary manual searches of the reference lists of included articles, Directory of Open Access Journals (DOAJ), metaRegister of Controlled Trials, Digital Dissertation (via UMI ProQuest), and Google Scholar were performed for studies to avoid omissions. The titles and abstracts of all identified studies were screened independently by two authors (A.K.C.Y. and H.H.C.) according to the eligibility criteria. Potentially eligible studies were retrieved for full‐text assessment by the two authors. Throughout the entire screening process, any disagreements between the two reviewers were resolved by discussion or consultation with a third reviewer (Z.S.). Inter‐reviewer reliability was calculated using Cohen's *κ*‐value.

### 2.5 Data Items and Collection

**FIGURE 1:**
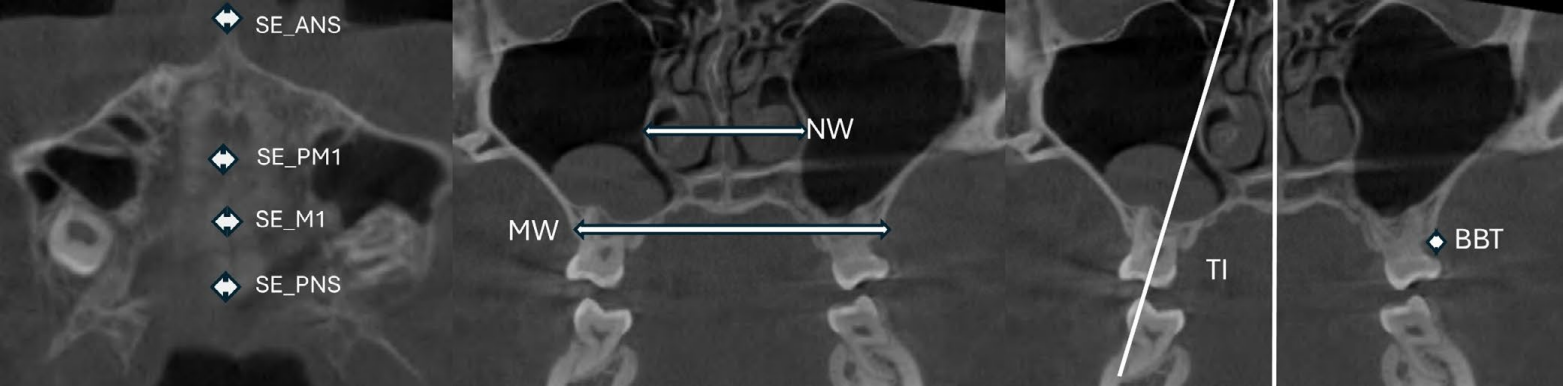
Illustration for SE, NW, MW, BBT and TI measurement.

The data extraction was conducted by the two reviewers independently from the full text of the included studies for their characteristics, including the author and publication year, study design, participants (number, gender, and age), intervention and comparators (appliances design and activation protocol), assessment settings (timepoint and parameters), and outcomes for immediate, short‐term, and long‐term effects, which were evaluated by retrieving the data of changes in skeletal and dentoalveolar bone at the observations within 1 week, within 6 months, and beyond 6 months from the baseline after maxillary expansion, respectively. The skeletal effects include the differences in the amount of expansion in (1) the mid‐palatal suture at the anterior nasal spine (SE_ANS), first premolar (SE_PM1), first molar (SE_M1) and posterior nasal spine (SE_PNS) regions; (2) the lateral limits of the nasal cavity at the first premolar (NW_PM1) and first molar (NW_M1) regions; and (3) the buccal surface of maxilla at the first premolar (MW_PM1) and first molar (MW_M1) regions. The dentoalveolar effects include the difference in the changes after expansion in (1) tooth inclination at the first premolar (TI_PM1) and first molar (TI_M1); and (2) buccal bone thickness at the first premolar (BBT_PM1) and first molar (BBT_M1) (Figure 1). For bilateral data, data of the left and right sides were averaged and used for quantitative data analysis.

### 2.6 Risk of Bias Assessment and Quality of Evidence

The risk of bias evaluation was performed using the Cochrane risk of bias tool (RoB 2) [30]. Quality of evidence was evaluated using GRADEpro software. Two authors assessed all included studies independently (A.K.C.Y. and H.H.C.). Any discrepancies were resolved by discussion with the third reviewer (Z.S.).

### 2.7 Summary Measures, Approach to Data Synthesis and Sensitivity Analysis

Corresponding to the included studies reported, evaluation time points were defined as follows: T0 indicates the baseline before expansion; T1 represents the final assessment conducted between immediately post‐expansion and 1 week thereafter; T2 is the last assessment taken after 1 week but within 6 months after expansion; T3 denotes the last assessment conducted after 6 months of expansion. The immediate, short‐term, and long‐term effects of BA or TB maxillary expansions were identified as changes in parameters for T1–T0 (immediate effect), T2–T0 (short‐term effect) and T3–T0 (long‐term effect), respectively. The BA expansion, which includes bone‐borne (BB) and hybrid tooth‐bone‐borne (TBB) expanders, is the intervention group, while the TB expansion is considered the comparator group. The mean changes in mid‐palatal suture (SE_ANS, SE_PM1, SE_M1 and SE_PNS), nasal width (NW_PM1 and NW_M1), maxillary width (MW_PM1 and MW_M1), anchored tooth inclination (TI_PM1 and T1_M1), and buccal bone thickness (BBT_PM1 and BBT_M1) were extracted for immediate, short‐term, and long‐term effects after expansion by two independent authors (A.K.C.Y. and H.H.C.) with their 95% confidence intervals in both groups. The difference in these outcome changes between BA and TB groups was subjected to meta‐analysis in Stata SE 14.0 (StataCorp, College station, Tx) for their comparative effects using the random‐effects model (DerSimonian & Laird approach). As the skeletal effect and dentoalveolar effect were assessed using multiple heterogeneous measurements, standardised mean differences (SMD) were utilised as the effect measure to enable comparisons across different but clinically related measurement tools.

The results were illustrated through forest plots and a summary‐of‐finding table. Statistical heterogeneity was evaluated using the *I* ^2^ values and Cochran's *Q* test (Chi^2^ test), and substantial heterogeneity between studies was quantified using the chi^2^ with a *p* value of < 0.10 or *I* ^2^ statistic of > 50%. The Grading of Recommendations Assessment, Development and Evaluation (GRADE) was employed to assess the quality of evidence and the strength of clinical recommendations based on five domains: risk of bias, inconsistency, indirectness, imprecision, and publication bias [33]. Additional analysis including sensitivity tests and influence analysis was performed in all the outcomes to analyse the outcome and the robustness of the result.

## 3 Results

### 3.1 Study Selection and Characteristics of Included Studies

**FIGURE 2:**
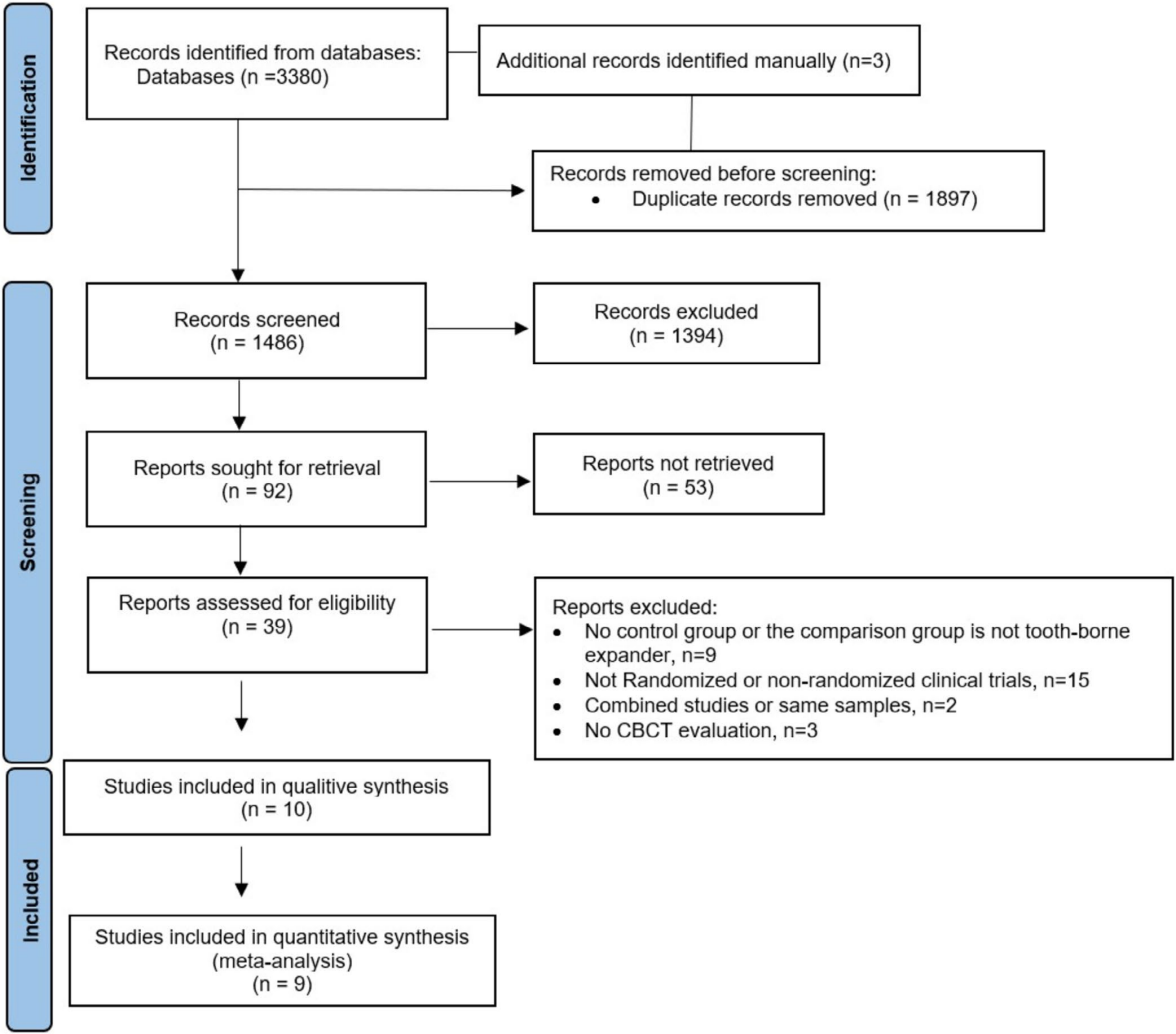
Flow diagram for the identification, screening, and inclusion of studies.

A total of 3383 records were yielded from the four electronic literature databases, with three more being identified manually. After removing the duplicates, 1486 records were assessed for their eligibility. Following title and abstract screening, 39 articles were reviewed in full text (Cohen's kappa value: 0.94) (Table S2). 29 articles were excluded: Fifteen study [5, 11, 15, 16, 34, 35, 36, 37, 38, 39, 40, 41, 42, 43, 44] were excluded for not fitting the CONSORT statement criteria of being an RCT [45]. Two studies [22, 46] were using the same patient sample and were combined for analysis. The study characteristics of another two studies [20, 47] revealed that the same patient samples were used. As a result, they were treated as one study, following the same procedure as above. After the selection process, ten randomised controlled trials [9, 10, 12, 13, 20, 21, 22, 48, 49, 50] were finally included in this systematic review. The study selection procedure is illustrated in the PRISMA flow diagram (Figure 2).

The ten eligible RCTs were conducted in university or private clinics across seven different countries (Brazil, Sweden, Netherlands, Turkey, South Korea, Canada, China) and included a total of 396 patients. The participants were randomly assigned to either the intervention group (BA expanders, *n* = 199) or the comparator group (TB expanders, *n* = 197). The average group size was approximately 19 patients in each trial. Except for one study [21] that did not report the number of males and females, the analysed sample included 158 males (43%) and 209 females (57%), with an average age of 13.3 years old. The descriptive statistics were presented in Table 2. Seven out of the ten studies [10, 12, 20, 22, 48, 49, 50] did not report the severity of transverse maxillary deficiency, nor did they mention the associated malocclusion features. In terms of appliance design, TB expanders were of the Hyrax type, while BA expanders varied across studies. Among the six studies using tooth‐bone‐borne (TBB, hybrid) expanders, four studies placed two screws paramedian to the mid‐palatal suture [10, 22, 48, 49] and two utilised four screws [12, 13]. For bone‐borne (BB), three studies used two screws [20, 21, 50] and one employed four screws [9]. All studies applied a similar rapid activation protocol, i.e., one to two quarters of a turn per day until overcorrection of maxillary posterior crossbite, except for one study that adopted one complete turn on the first day [10]. For the groups using hybrid or TB expansion, maxillary first molars served as anchors, with either palatal arms or bands on the maxillary first premolars. Retention periods varied among studies. Four studies [10, 12, 13, 49] maintained the appliance passively for 3 months, four [20, 21, 22, 50] for 6 months, and one [48] for 11 months.

**TABLE 2:** Descriptive data of the selected studies.

Author and year (country)	Study design	Participants	Intervention‐appliance design	Intervention‐activation protocol	Intervention‐duration	Assessment‐time point	Assessment‐approach	Assessment‐parameters	Outcomes
Lagravere MO, et al. 2010 (Canada)	Parallel	BA: *n* = 21 (Male, *n* = 8; Female, *n* = 13; Age: 14.24 ± 1.32) TB: *n* = 21 (Male, *n* = 6; Female, *n* = 15; Age: 14.05 ± 1.35) Severity of transverse maxillary deficiency was not mentioned	BA: 2 screws paramedian to the mid‐palatal suture; TB: bands on upper 1st molars and premolars	BA: 1× every other day until overcorrection is reached; TB: 2×/day until overcorrection is reached	Activation: Not mentioned Retention:6 months	T0, T1, T2, T3 (T0): Baseline (T1): Immediately after expansion (T2): within 6 months after expansion (T3): 12 months after placement, before fixed appliance bonding	CBCT (NewTom 3G; 110 kV, 6.19mAs, voxel size: 0.25 mm, 8 mm aluminium filtration); Measurement using AMIRA	‐ SE_PNS ‐ MW_PM1 ‐ MW_M1 ‐ Premolar and molar tipping (angulation and distance measured through the pulp chamber level and root apex of the 1st premolars and 1st molars) ‐ angles for symmetrical changes (from pulp chamber of 1st molar to infraorbital foramen on both sides) ‐width changes in infraorbital foramen and mental foramen	*Immediate effect (T1‐T0)* 1. There was more maxillary first premolar buccal crown tipping in tooth‐borne expansion (TB) group than bone‐anchored (BA) expanders (*p* = 0.003) 2. Crown expansion was greater than apical and skeletal expansion with both appliances 3. The control group showed minimal changes over the 6 months *Short‐term effect (T2‐T0)* 1. Significantly more buccal crown tipping in tooth‐borne expansion group than bone‐anchored expanders (*p* < 0.001), while there was no significant difference in sutural expansion, increase in maxillary width and molar tipping (*p* > 0.05) *Long‐term effect (T3‐T0)* 1. All groups had similar expansion angels, indicating expansion was symmetrical 2. Both groups had long‐term expansion at the 1st molar and 1st premolar crowns, root apex, alveolus level, and root of central incisor

Gunyuz Toklu, et al. 2015 (Turkey)	Parallel	BA: *n* = 12 (Male, *n* = 6; Female, *n* = 6; Age: 13.8 ± 2.2) TB: *n* = 13 (Male, *n* = 5; Female, *n* = 8, Age: 14.3 ± 2.3) Severity of transverse maxillary deficiency was not mentioned	BA: 2 screws paramedian to the mid‐palatal suture. Molar bands were fitted to the maxillary right and left first molars. TB: bands on upper 1st molars and premolars	BA and TB: 2×/day until palatal cusps of upper molar touch lower molar buccal cusps	Activation: 20 days Retention: 3 months, followed by a transpalatal arch placed afterwards	T0, T2 (T0): Baseline (T2): 3 months after expansion	CBCT (120 kV, 3.8 mA, voxel size: 0.2 mm, axial slice thickness: 0.3 mm, FOV: 20 × 25 cm, exposure time: 40 s); Measurement using Mimics	‐ SE_PNS ‐ NW_M1 ‐ MW_M1 ‐ TI_PM1 ‐ TI_M1 ‐ BBT_PM1 ‐ BBT_M1 ‐ palatal bone thickness for both sides ‐ molar and premolar alveolus inclination for both sides ‐ absolute dental tipping at 1st molar and 1st premolar for both sides	*Short‐term effect (T2‐T0)* 1. Both groups revealed significant changes in skeletal structures and increase in interdental distances. 2. The increase in distance between the 1st premolar increased more in tooth‐borne (TB) expansion group (7.5 ± 4.2 mm) than the bone‐anchored (BA) group (3.2 ± 2.6 mm) (*p* < 0.05) 3. Similar reductions in buccal bone thickness (BBT_M1) and increase in palatal bone thickness at the 1st molar (*p* > 0.005) in both groups 4. There was decrease in 1st premolar buccal bone thickness (BBT_PM1) in TB group, but no change in BA group (*p* < 0.05) (TB: 0.8 ± 0.65 mm; BA: 0.009 ± 0.59 mm) 5. Significantly more 1st premolar tipping in TB group (−2.33° ± 3.03°) than BA group (0.45° ± 2.51°) (*p* > 0.05), while there was no significant difference in 1st molar tipping (TB: −2.96° ± 3.47°; BA: −2.89° ± 4.90°) (*p* > 0.05) 6. No significant difference in absolute dental tipping in 1st molar and 1st premolar for both groups. (*p* > 0.05)
Celenk‐Koca, et al. 2018 (Netherlands)	Parallel	BA: *n* = 20 (Male, *n* = 7; Female, *n* = 13; Age: 13.81 ± 1.23) TB: *n* = 20 (Male, *n* = 8; Female, *n* = 12; Age: 13.84 ± 1.36) Included samples planned to have 8 mm screw activation	BA: 4 screws used. Two anterior screws are between 1st and 2nd premolars. Another two screws are between 2nd premolars and 1st molars TB: casted, covering from 1st premolar to 1st molar with palatal armas on 2nd molar	BA, TB: 2×/day until overcorrection is reached	Activation: 19.7 ± 3.8 days Retention: Not mentioned	T0, T2 (T0): Baseline (T2): within 6 months after expansion	CBCT (70 kV, 10 mA, voxel size: 76‐μm, FOV: 8 cm × 8 cm, exposure time: 32.5 s); Measurement using OsiriX Imaging version 6.5	‐ SE_ANS ‐ SE_PM1 ‐ SE_M1 ‐ NW_M1 ‐ TI_PM1 ‐ TI_M1 ‐ BBT_PM1 ‐ BBT_M1 ‐ root length	*Short‐term effect (T2‐T0)* 1. Bone anchored (BA) expansion has greater amount (2.5 times more) of mid‐palatal sutural opening than tooth‐borne (TB) expansion group both anteriorly and posteriorly 2. The sutural expansion acted for 28% and 70% at the 1st premolar, 26% and 68% at the 1st molar of the total transverse width increase in both groups 3. Most patients exhibited a triangular shaped sutural opening which was wider anteriorly 4. More reduction in the buccal bone thickness of the 1st premolar (−0.29 ± 0.2 mm) and 1st molar (−0.24 ± 0.2 mm) in the TB group, compared to the BA group (1st premolar: −0.04 ± 0.2 mm; 1st molar: −0.10 ± 0.1 mm) (*p* < 0.05) 5. There was greater buccal inclination of the 1st premolar (4.5° ± 3.0°) and 1st molar (3.9° ± 3.4°) in the TB group, compared to the BA group (1st premolar: 0.6° ± 2.4°; 1st molar: 1.3° ± 2.1°) (*p* < 0.05) 6. No significant difference detected for root length measurement in both groups
Davami K, et al. 2020 (Canada)	Parallel	BA: *n* = 14; TB: *n* = 15; Male, Female: non mentioned Age: 11–17 Included samples had minimum of 5 mm maxillary constriction (cusp‐to‐fossa)	BA: 2 screws paramedian to the mid‐palatal suture (Dresden‐type: temporary anchorage device, TAD on one side; and a shortened implant on the other side) TB: bands on upper 1st molars and premolars	BA: 1×/day until overcorrection (20%) is reached TB: 2×/day until overcorrection is reached	Activation: Not mentioned Retention: 4–6 months, followed by a transpalatal arch placed afterwards	T0, T3 (T0): Baseline (T3): two or more years after expansion	CBCT (120 kV, 5 mA, voxel size: 0.3 mm FOV: 13 cm × 16 cm, exposure time: 8.9 s, 8 mm aluminium filtration); Measurement using AVIZO 9 Lite	‐ SE_ANS ‐ SE_PNS ‐ displacement of alveolar bone at molar, premolar and canine area ‐ displacement of pulp tip at molar, premolar and canine area ‐ displacement of molar root apex tip	*Long‐term effect (T3‐T0)* 1. The anterior and posterior skeletal expansion in both groups (tooth‐borne, TB and bone‐anchored, BA expansion) did not show significant differences (Posteriorly, BA: 1.91 mm, TB: 1.96 mm; Anteriorly, BA: 1.16 mm, TB: 0.52 mm) (*p* > 0.05) 2. The lateral crown displacement measured at the pulp tip of molar, premolar and canine did not show significant difference in changes between two groups. (*p* > 0.05) 3. No significant difference in alveolar bone displacement between two groups at the molar, premolar and canine region (*p* > 0.05)
Lagravere MO, et al. 2020 (Canada)	parallel	BA: *n* = 17 (Male, *n* = 7; Female, *n* = 10; Age: 14.1 ± 1.6) TB: *n* = 17 (Male, *n* = 8; Female, *n* = 9; Age: 13.7 ± 1.1) Severity of transverse maxillary deficiency was not mentioned	BA: 2 screws paramedian to the mid‐palatal suture (Dresden‐type: temporary anchorage device, TAD on one side; and a shortened implant on the other side) TB: bands on upper 1st molars and premolars	BA: 1×/day until overcorrection (20%) is reached TB: 2×/day until overcorrection is reached	Activation: At least 5 mm activation Retention with the appliance kept in place sealed with light‐cured composite for additional 4–6 months	T0, T2 (T0): Baseline (T2): within 6 months after expansion	CBCT (120 kV, 5 mA, voxel size: 0.3 mm, FOV: 13 cm × 16 cm, exposure time: 9 s, 8 mm aluminium filtration); Measurement using AVIZO 8.1 software	‐ SE_PNS ‐ inter‐molar and inter‐premolar width, measured between the tips of pulp‐horns ‐ dental root widths at 1st molar and 1st premolar ‐ intermolar buccal alveolar width	*Short‐term effect (T2‐T0)* 1. Both groups (bone anchored, BA and tooth‐borne, TB) demonstrated significant skeletal expansion (BA: 1.31 mm; TB: 1.27 mm) and dental expansion with buccal crown tipping in both groups (TB: 5.66 mm; BA: 4.17 mm), (both *p* < 0.01) 2. No significant difference in skeletal and dental expansion was found both groups (*p* > 0.05) 3. BA expansion group revealed a lower ratio of dental to skeletal expansion (20:80) than TB expansion (40:60) group. 4. TB expansion showed a symmetrical pattern, while the BA expansion revealed an asymmetrical pattern in relation to the mid‐sagittal plane 5. Significant forward movement of 1st premolar and molar (1.5 mm, *p* < 0.05) and vertical dental extrusion (premolar: 1.5 mm, molar: 1.8 mm, *p* < 0.05) in TB expansion group, while there were no significant dental vertical or antero‐posterior changes in BA group

Bazargani F, et al. 2021 (Sweden)	Parallel	BA: *n* = 26 (Male, *n* = 13; Female, *n* = 13; Age: 9.5 ± 1.2) TB: *n* = 26 (Male, *n* = 13; Female, *n* = 13; Age: 9.3 ± 1.3) Severity of transverse maxillary deficiency was not mentioned	BA: 2 screws paramedian to the mid‐palatal suture. Molar bands were fitted to the maxillary right and left first molars, with palatal arms extending to right and left first premolars. TB: bands on upper 1st molars, with palatal arms	BA, TB: 2×/day until overcorrection is reached	Activation: Not mentioned Retention with the appliance kept in place for 6 months	T0, T1, T3 (T0): Baseline (T1): Immediately after expansion (T3): 1 year after expansion	CBCT (Accuitomo 170, Kyoto Japan, 90 kV, 4 mA, voxel size: 0.25 mm FOV: 14 cm × 10 cm axial slice thickness: 0.25 mm); Measurement using OsiriX Imaging version 6.5	‐ SE_ANS ‐ SE_PM1 ‐ SE_M1 ‐ SE_PNS ‐ NW_PM1 ‐ NW_M1 ‐ NW at ANS and PNS region ‐ TI_M1 ‐ BBT_M1 ‐ tipping of alveolar bone ‐ dental expansion measured on plaster models	*Immediate effect (T1‐T0)* 1. Skeletal expansion at the nasal cavity was significant greater (1 mm, *p* = 0.0001) in the bone‐anchored (BA) expansion group, compared to the tooth‐borne (TB) group, though this might not be clinically significant 2. Magnitude of expansion at the nasal cavity level was 2 times greater in BA expansion group than in TB group. (*p* = 0.0001) 3. TB group revealed more 1st molar buccal crown tipping than BA group (2.2°, *p* = 0.009) 4. Dental expansion, and alveolar bending showed no significant difference between the two groups *Long‐term effect (T3‐T0)* The stability of treatment result (1‐year post‐expansion) was the same between the two groups
Garib D, et al. 2021 (Brazil)	Parallel	BA: *n* = 18 (Male, *n* = 10; Female, *n* = 8; Age: 10.8 ± 1.04) TB: *n* = 14 (Male, *n* = 8; Female, *n* = 6; Age: 11.4 ± 1.26) Severity of transverse maxillary deficiency was not mentioned	BA: 2 screws paramedian to the mid‐palatal suture. Molar bands were fitted to the maxillary right and left first molars, with palatal arms extending to right and left first premolars. TB: bands on upper 1st molars, with palatal arms	BA, TB: 2×/day until overcorrection is reached	Activation: 14 days (5.6 mm expansion) Retention with the appliance kept in place for 11 months	T0, T3 (T0): Baseline (T3): 1 year after expander removal	CBCT (N/A) Measurement using NemoScan	‐ NW_M1 ‐ MW_M1 ‐ 1st molar crown inclination (angle between lines passing through the buccal and lingual cusp tips of 1st molars) ‐ buccal alveolar crest width‐ intermolar, interpremolar and intercanine width‐ arch length and arch perimeter	*Long‐term effect (T3‐T0)* 1. BA expansion group demonstrated greater increases in nasal cavity width (1.15 mm, *p* = 0.04) 2. BA expansion group demonstrated greater increases maxillary width (0.83 mm, *p* = 0.041) 3. BA expansion group demonstrated greater increases in buccal alveolar crest width (1.19 mm, *p* = 0.023) 4. Similar dental effects with no significant differences in intermolar, interpremolar or intercanine width in both groups 5. Similar arch size and shape changes for both groups
Jia H, et al. 2021 (China)	Parallel	BA: *n* = 30 (Male, *n* = 9; Female, *n* = 21; Age: 15.1 ± 1.6) TB: *n* = 30 (Male, *n* = 12; Female, *n* = 18; Age: 14.8 ± 1.5) Included samples need to have more than 5 mm maxillary expansion	BA: 4 screws paramedian to the mid‐palatal suture. Molar bands were fitted to the maxillary right and left first molars. TB: bands on upper 1st molars and premolars	BA, TB: 2×/day until overcorrection is reached	Activation: Not mentioned Retention: with the appliance kept in place for 3 months, after which patients continued with fixed appliance	T0, T1 (T0): Baseline (T1): 1 week after expansion	CBCT (KaVo 3D eXam device, USA, 120 kV, 18.54 mA, voxel size: 0.3 mm, exposure time: 8.9 s); Measurement using Invivo 5	‐ success rate of suture opening ‐ SE_ANS ‐ SE_PM1 ‐ SE_M1 ‐ SE_PNS ‐ NW_PM1 ‐ NW_M1 ‐ MW_PM1 ‐ MW_M1 ‐ TI_PM1 ‐ TI_M1 ‐ Alveolar height at 1st molar region‐ Skeletal to dental expansion ratio at the 1st molar level (sutural expansion/inter‐crown width of 1st molar)	*Immediate effect (T1‐T0)* 1. success rate of mid‐palatal sutural opening in bone‐anchored (BA) expansion group (100%) was greater than the tooth‐borne (TB) expansion group (86.7%) 2. Palatal expansion was greater in BA group (3.82 mm) than TB group (2.20 mm) (*p* < 0.01) 3. The skeletal to dental expansion ratio in BA group (61.4%) is around 2 times higher than that in TB group (32.3%) (*p* < 0.01) 4. Both BA and TB groups showed reduction in alveolar bone height, but there was less reduction in buccal alveolar bone height and buccal tipping at the 1st molars in BA group, compared to the TB group (*p* < 0.01)

Chun JH, et al. 2022 (South Korea)	Parallel	BA: *n* = 19 (Male, *n* = 8; Female, *n* = 12; Age: 14.1 ± 4.2) TB: *n* = 17 (Male, *n* = 6; Female, *n* = 14; Age: 14.0 ± 4.5) Severity of transverse maxillary deficiency was not mentioned	BA: 4 screws paramedian to the mid‐palatal suture, along with bands on upper 1st molars and premolars. Bands were fitted to the maxillary right and left first molars and premolars. TB: bands on upper 1st molars and premolars	BA, TB: 1×/day until overcorrection is reached	Activation: 35 times for both BA and TB (7.0 mm expansion) Retention: with the appliance kept in place for 3 months, after which patients continued with fixed appliance	T0, T1, T2 (T0): Baseline (T1): Immediately after expansion (T2): 3 months after expansion	CBCT (Alphard 3030, Kyoto, Japan; 80 kV, 3.0 mA; voxel size: 0.39 mm; FOV: 20 cm × 20 cm; exposure time: 17 s) Measurement using Invivo 5 and Dolphin Imaging and Management Solutions	‐ SE_ANS ‐ SE_PNS ‐ NW_PM1 ‐ NW_M1 ‐ MW_PM1 ‐ MW_M1 ‐ TI_M1 ‐ BBT_PM1 ‐ BBT_M1 ‐ Frequency of mid‐palatal suture opening‐ interdental width between the buccal cusp tips at the 1st molar and 1st premolar ‐ palatal bone thickness	*Immediate effect (T1‐T0)* 1. Tooth‐borne expansion (TB) showed midpalatal sutural opening with 90% success rate, while BA group in 95% 2. Both groups exhibited significant triangular basal bone expansion (T1‐T0), with relapse in the skeletal effect during the consolidation period (T2‐T0) 2. Greater increase in nasal width at the 1st molar (BA: 2.88 ± 0.82 mm; TB: 1.95 ± 0.81 mm, *p* = 0.002) and greater palatine foramen (BA: 2.70 ± 1.18 mm; TB: 1.84 ± 1.31 mm, *p* = 0.048) in the BA expansion group 4. Similar dentoalveolar changes (interdental width) in both groups, except maxillary width 5. The BA group revealed greater increase in maxillary width at the 1st molar region (BA: 3.31 ± 0.82 mm; 2.59 ± 1.17 mm, *p* = 0.038) 6. More stable positioning of the anchored teeth in BA group regarding the bone thickness changes at the 1st premolar and 1st molar region *Short‐term effect (T2‐T0)* 1. Greater increase in the nasal width (BA: 2.19 ± 0.84, TB: 1.55 ± 1.02, *p* = 0.045) and maxillary width (BA: 3.99 ± 2.46 mm; TB: 2.16 ± 2.00 mm, *p* = 0.019) at the 1st premolar 2. Both groups show reduction in buccal bone thickness, with BA group less reduction (BA: −0.45 ± 0.56 mm; TB: −0.91 ± 0.40 mm, *p* = 0.007) 3. With the same amount of expansion, the BA group revealed lower reduction in the buccal bone thickness after the consolidation period 3. Less buccal displacement of the anchored teeth was observed in BA group during the consolidation period
Pasqua BPM, et al. 2022 (Brazil)	parallel	BA: *n* = 21 (Male: *n* = 9; Female: *n* = 12; Age: 13.3 ± 1.3) TB: *n* = 21 (Male: *n* = 16; Female: *n* = 5; Age: 13.2 ± 1.4) Severity of transverse maxillary deficiency was not mentioned	BA: 2 screws paramedian to the mid‐palatal suture, anterior region of palate, posterior to 3rd palatal rugae line. Molar bands were fitted to the maxillary right and left first molars. TB: bands on upper 1st molars and premolars	BA, TB: 4× on the 1st activation day. After that, 2×/day until overcorrection is reached	Activation: Not mentioned Retention with the appliance kept in place for 3 months	T0, T2 (T0): Baseline (T2): 3 months after RME	CBCT (iCAT, Hatfield, Pennsylvania; 120 kV; 18 mA; exposure time: 8.9 s; voxel size: 0.2 mm; FOV: 16 cm × 60 mm); Measurement using Dolphin Imaging and Management Solutions	‐ NW_PM1 ‐ NW_M1 ‐ MW_PM1 ‐ MW_M1 ‐ TI_PM1 ‐ TI_M1 ‐ nasal cavity width ‐ intercrown distance of the 1st premolars and 1st molars ‐ distance between apices of 1st premolars and 1st molars	*Short‐term effect (T2‐T0)* 1. At the premolar region, greater increase in nasal cavity width (1.5 mm, *p* = 0.004), nasal floor width (NW_PM1) (1.4 mm, *p* = 0.019) and maxillary width (1.1 mm, *p* = 0.022) at the premolar region in the bone‐anchored (BA) expansion group, than tooth‐borne (TB) group 2. At the molar region, greater increase in nasal cavity width (0.9 mm, *p* = 0.005) and maxillary width (0.9 mm, *p* = 0.042) 3. Increase in the distance between 1st premolar crowns was greater in TB group (1.8 mm, *p* = 0.006), with no differences between the apices of the premolar roots (*p* > 0.005) 4. More buccal tipping of 1st premolar (3.2°, *p* = 0.009) in TB group, with minor tooth inclination changes in BA expansion group 5. No differences in dental changes at the 1st molars in both groups

All studies used CBCT images to evaluate the skeletal and dentoalveolar effects, with baseline and post‐expansion measurements recorded. Among all the included studies, only nine studies [9, 10, 12, 13, 20, 21, 22, 49, 50] provided detailed information on scanning settings. Various software was used for data measurement and analysis, including AMIRA [20], Mimics [49], OsiriX Imaging [9, 22], AVIZO 9 Lite [21], Nemoscan [48], Invivo 5 [12, 13], and Dolphin imaging [10, 12]. The timing of assessment also varied across studies: three trials [12, 20, 22] measured outcomes immediately or within one week after expander removal (immediate effect, T1), six trials [9, 10, 12, 20, 49, 50] assessed effects between 1 week and 6 months post‐expansion (short‐term effect, T2), and four trials [20, 21, 22, 48] conducted evaluations beyond 6 months (long‐term effect, T3).

### 3.2 Risk of Bias Assessments

**FIGURE 3:**
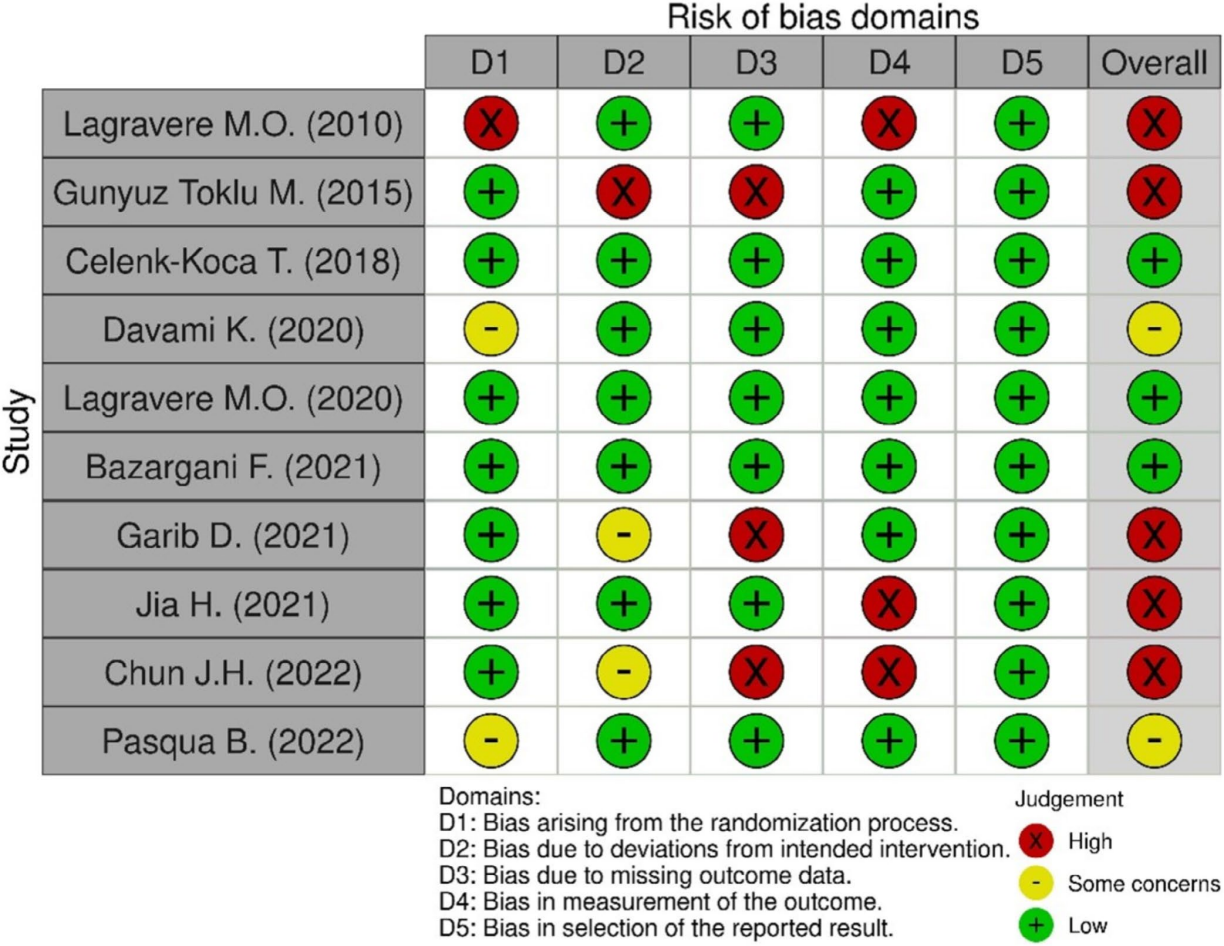
Risks of Bias summary for included studies (Cochrane RoB 2 tool).

The overall risk of bias of the included studies ranged from low (two studies) to some concerns (two studies) to high (five studies) (Figure 3). The domains of concern with a high risk of bias included randomization (one study [20] lacked randomization and concealed allocation), dropout description (one study [49] excluded the sample due to loss of mini‐screws), missing outcome data (three studies [12, 49, 51] excluded samples with mini‐screws failure or maxillary expansion or loss of follow‐up) and bias in measurement of outcome (three studies [12, 13, 20] had no blinding for measurement). As for the blinding strategies among the included studies, one study [10] performed blinding by removing the appliances during the CBCT acquisition. Three studies [12, 13, 20] did not perform any blinding, while the remaining six studies [9, 21, 22, 48, 49] only performed blinding by de‐identifying CBCT assessment.

### 3.3 Results of Meta‐Analyses at Different Time Points

Meta‐analysis utilised quantitative data from the 9 studies [9, 10, 12, 13, 20, 21, 22, 48, 49]. Different studies conducted various measurements to evaluate sutural expansion. One study [22] measured the superior and inferior parts of mid‐palatal sutural width separately, and these measurements were combined into one value for comparability. Two studies [9, 21] evaluated the changes in incisive foramen width, which were considered representative of sutural expansion at the anterior nasal spine (SE_ANS). A similar approach was applied for posterior nasal spine expansion (SE_PNS), with measurements derived from two studies [12, 21] on greater palatine foramen, and two studies [20, 49] on lateral pterygoid plates. Sutural expansion was assessed at different time points: four studies [12, 13, 20, 22] measured the amount of sutural expansion at T1, four studies [9, 12, 49, 50] at T2 and three studies [20, 21, 22] at T3.

Nasal width (NW) changes were measured between the inner lateral limits of the nasal cavity, while maxillary width (MW) changes were assessed based on the distance change between right and left points on the maxillary basal bone curve. Both nasal and maxillary width were measured at two regions: the upper 1st premolar (NW_PM1, MW_PM1) and the upper 1st molar (NW_M1, MW_M1). NW changes were evaluated by three studies [12, 13, 22] at T1, four studies [9, 10, 12, 49] at T2, and two studies [22, 48] at T3. MW changes were assessed by three studies [12, 13, 20] at T1, four studies [10, 12, 20, 49] at T2 and two studies [20, 48] at T3.

Tooth inclination changes for premolar (TI_PM1) and molar (TI_M1) were calculated based on the angle between the tooth's long axis and a reference plane (either the sagittal plane or the horizontal plane). One study [48], which measured the angle between the buccal and lingual cusp tips on both sides, was excluded due to a lack of comparability. Two studies [9, 13] employed the horizontal plane as reference, whereas four studies [10, 12, 22, 49] used the mid‐sagittal plane, necessitating angle transformations for comparability. Tooth inclination changes were evaluated at T1 in three studies [12, 13, 22], T2 in five studies [9, 10, 12, 49, 50] and T3 in one study [22]. Reduced tooth inclination (tipping) indicated less dentoalveolar effect and a more favourable treatment effect for the BA group. Five studies [10, 13, 20, 22, 49] measured inclination on both the left and right sides of the 1st premolar and 1st molar, with data extracted for further analysis.

Changes in the marginal buccal bone thickness at the cementoenamel junction or furcation of the upper 1st premolar (BBT_PM1) and molar region (BBT_M1) were assessed in the axial section. The assessment occurred at T1 in two studies [12, 22], T2 in one study [12] and T3 in one study [22]. Two studies [22, 49] measured the left and right sides of 1st molar, while others [12, 49] assessed the mesial and distal roots. These data were combined and processed for further analysis.

#### 3.3.1 Immediate Effects

One important skeletal effect of maxillary expanders was suture expansion (SE), which had been examined at four regions across the anteroposterior plane [12, 13, 20, 22]. BA expander significantly outperformed TB in SE_ANS (SMD: 0.77, 95% CI: 0.43–1.11), SE_PM1 (SMD: 1.04, 95% CI: 0.64–1.43), SE_M1 (SMD: 1.50, 95% CI: 1.05–1.94), and SE_PNS (SMD: 0.87, 95% CI: 0.55–1.18). However, high heterogeneity of studies was detected at the SE_M1 (*I* ^2^ = 94.7%) and SE_PNS (*I* ^2^ = 89.4%), indicating cautious attention is needed to pay when interpreting these results. In terms of effects on nasal and maxillary widths, BA expanders produced a greater increase in NW_M1 (SMD: 0.85, 95% CI: 0.51–1.19), NW_PM1 (SMD: 0.60, 95% CI: 0.27–0.94), MW_M1 (SMD: 1.06, 95% CI: 0.62–1.49), and MW_PM1 (SMD: 0.51, 95% CI: 0.16–0.86) compared to TB expanders. Moderate heterogeneity was observed for measurements in the molar region: NW_M1 (*I* ^2^ = 60.5%) and MW_M1 (*I* ^2^ = 43.0%), while the premolar region (NW_PM1 and MW_PM1) had significant heterogeneity (*I* ^2^ > 80%) (Figure S1).

In terms of dentoalveolar effects, only one study [13] evaluated the first premolar region, indicating TB resulted in a greater increase in tooth inclination(TI_PM1; SMD: −1.52, 95% CI: −2.10 to −0.94) and a greater reduction in buccal bone thickness (BBT_PM1; SMD: 0.83, 95% CI: 0.15–1.52) compared to the BA type. Three studies [12, 13, 22] assessed the dentoalveolar effects of the molar region, suggesting there was no significant standard mean difference in the TI_M1 (SMD: −0.30, 95% CI: −0.63 to 0.02) and BBT_M1 (SMD: −0.16, 95% CI: −0.57 to 0.26) between the two groups (Figures S2 and S3).

#### 3.3.2 Short‐Term Effects

Short‐term skeletal effects of BA and TB were compared in terms of suture expansion, nasal width, and maxillary width. Only one study [9] evaluated SE_PM1 and SE_M1, showing BA expander outperformed TB (SE_PM1, SMD: 2.29, 95% CI: 1.48–3.11; SE_M1, SMD: 2.04, 95% CI: 1.26–2.82). Pooled results indicated that BA expanders produced greater expansion at the anterior nasal spine (SE_ANS; SMD: 1.12, 95% CI: 0.61–1.63, *I* ^2^ = 89.8%) [9, 12] and posterior nasal spine (SE_PNS; SMD: 0.27, 95% CI: −0.13 to 0.66, *I* ^2^ = 64.6%) [12, 20, 49]. As for nasal width, the pooled analyses indicated BA types had greater expansion on NW_PM1 (SMD: 0.97, 95% CI: 0.58–1.36, *I* ^2^ = 75.0%) [10, 12, 38] and NW_M1 (SMD: 1.18, 95% CI: 0.80–1.57, *I* ^2^ = 92.2%) [9, 10, 12, 49] compared to the TB expanders. Similarly, for maxillary width, greater changes were observed in MW_PM1 [10, 12, 20] (SMD: 0.59, 95% CI: 0.18–1.01, *I* ^2^ = 96%) and MW_M1 [10, 12, 20, 49] (SMD: 0.46, 95% CI: 0.11–0.82, *I* ^2^ = 90.0%) (Figure S4).

Analysis of four studies [9, 10, 12, 49] revealed that BA expanders resulted in significantly less TI_PM1 (SMD: −1.77, 95% CI: −2.25 to −1.29, *I* ^2^ = 89.9%) compared to TB expanders. However, no significant difference was found in TI_M1 between the two groups (SMD: −0.22, 95% CI: −0.57 to 0.13, *I* ^2^ = 90.7%) (Figure S5). Additionally, three studies [9, 12, 49] indicated that BA expansion led to less reduction in the BBT_PM1 (SMD: 1.29, 95% CI: 0.86 to 1.73, *I* ^2^ = 0.0%) and BBT_M1 (SMD: 0.68, 95% CI: 0.28 to 1.09, *I* ^2^ = 0.0%) (Figure S6).

#### 3.3.3 Long‐Term Effects

Pooled analyses of studies showed no significant differences in sutural expansion between BA and TB groups for both anterior [21, 22] (SE_ANS; SMD: −0.28, 95% CI: −0.72 to 0.16, *I* ^2^ = 0.0%) and posterior nasal spine regions [20, 21, 22] (SE_PNS; SMD: −0.13, 95% CI: −0.48 to 0.23, *I* ^2^ = 0.0%). One study [22] revealed that at the premolar region, there was greater nasal width, with less maxillary width. Pooled studies indicated that the BA group had greater nasal width at the molar region [22, 48] (NW_MI; SMD: 0.63, 95% CI: 0.19–1.08, *I* ^2^ = 41.5%) and maxillary width [20, 48] (MW_MI; SMD: 0.21, 95% CI: −0.26 to 0.67, *I* ^2^ = 60.7%) (Figure S7).

Additionally, one study [22] reported that BA expansion had less buccal tipping (TI_M1; SMD: −0.72, 95% CI: −1.28 to −0.15) than TB expansion, while no significant difference was observed in molar buccal bone thickness between the two types (BBT_M1; SMD: 0.00, 95% CI: −0.54 to 0.54) (Figure S7).

#### 3.3.4 Additional Analyses—Influence Analysis

In general, the influence analysis revealed relatively robust results (Figures S8–S11), while some effect estimates were still sensitive to the exclusion of certain studies. For example, it is notable to mention that removing one of the outlier studies (Lagravere MO et al. [20]) for the short‐term effect of maxillary width change at the premolar region caused a significant change in the overall SMD. Specifically, the SMD shifted from 0.59 (95% CI: 0.18–1.01, Figure S5) to 1.52 (95% CI: 0.96–2.09, Figure S9). Noteworthy, Pasqua et al. [10] also significantly influence the heterogeneity of nasal width and maxillary width changes in the short‐term effect, which was very high (Figure S9). To mitigate the effects of its heterogeneity, a further sensitivity analysis removing the outliers (Pasqua et al. [10]) was conducted (Figure S11). The pooled result showed that heterogeneity was greatly reduced in most analyses, except maxillary width at the premolar region (MW_PM1). The overall result of the nasal width change remains the same, with a significantly greater effect at the hybrid/bone‐borne group (Premolar—SMD: 0.61, 95% CI: 0.15–1.07; Molar—SMD: 0.78, 95% CI: 0.37–1.19) and no heterogeneity at the premolar region (*I* ^2^ = 0.0%, *p* = 0.810), and moderate heterogeneity at the molar region (*I* ^2^ = 58.8%, *p* = 0.088). Maxillary width changes at the premolar and molar regions revealed that there was no significant difference between the two groups (Premolar—SMD: −0.00, 95% CI: −0.46 to 0.45; Molar—SMD: 0.01, 95% CI: −0.38 to 0.40), with slightly reduced heterogeneity at the premolar region (*I* ^2^ = 81.6%, *p* = 0.020) and no heterogeneity at the molar region (*I* ^2^ = 21%, *p* = 0.282).

### 3.4 GRADE Assessment

The GRADE scores are summarised in Table 3. Six outcomes were assessed across different time points. Measurements of the immediate effect (SE_ANS, MW_M1, BBT_PM1, BBT_M1), the short‐term effect (NW_PM1 and NW_M1, BBT_PM1), and the long‐term effect (SE_ANS, NW_M1) received a moderate quality rating. However, many other outcomes were rated as low or very low in quality at all time points, which was affected by potential high risks of bias and inconsistency of some of the studies [12, 13, 20, 48, 49].

**TABLE 3:** GRADE quality assessment and summary of findings across studies.

Quality assessment						No. of patients		Summary of findings	
No. of studies	Study design	Risk of bias	Inconsistency	Indirectness	Imprecision	Tooth‐bone‐borne/bone‐borne expander	Tooth‐borne expander (control group)	Absolute (95% CI)	Quality
Immediate effect									
Sutural Expansion at Anterior Nasal Spine (ANS) region, SE_ANS—immediate effect									
3	Randomised trials	Serious^a^, ^b^	Not serious	Not serious	Not serious	75	73	SMD **0.77** **(*p*<0.001)** (0.43 to 1.11)	⊕⊕⊕○ Moderate
Sutural Expansion at Poterior Nasal Spine (PNS) region, SE_PNS—immediate effect									
4	Randomised trials	Serious^a^, ^c^	Very serious	Not serious	Not serious	96	94	SMD **0.87** **(*p*<0.001)** (0.55 to 1.18)	⊕○○○ Very low
Nasal Width, at premolar region, NW_PM1—immediate effect									
3	Randomised trials	Serious^a^, ^b^	Very serious	Not serious	Not serious	75	73	SMD **0.60** **(*p*<0.001)** (0.27 to 0.94)	⊕○○○ Very low
Nasal Width at molar region, NW_M1—immediate effect									
3	Randomised trials	Serious^a^, ^b^	Serious	Not serious	Not serious	75	73	SMD **0.85** **(*p*<0.001)** (0.51 to 1.19)	⊕⊕○○ Low
Maxillary Width at premolar region, MW_PM1—immediate effect									
3	Randomised trials	Serious^a^, ^b^, ^c^	Very serious	Not serious	Not serious	70	68	SMD **0.51** **(*p*=0.004)** (0.16 to 0.86)	⊕○○○ Very low
Maxillary Width at molar region, MW_M1—immediate effect									
2	Randomised trials	Serious^a^, ^b^	Not serious	Not serious	Not serious	49	47	SMD **1.06** **(*p*<0.001)** (0.62 to 1.49)	⊕⊕⊕○ Moderate
Molar buccal tipping, TI_M1—immediate effect									
2	Randomised trials	Serious^a^, ^b^	Serious	Not serious	Serious	75	73	SMD −0.30 (*p*=0.070) (−0.63 to 0.02)	⊕○○○ Very low
Premolar Buccal bone thickness, BBT_PM1—immediate effect									
1	Randomised trials	Serious^a^, ^b^	Not serious	Not serious	Not serious	45	43	SMD **0.83** **(*p*=0.018)** (0.15 to 1.52)	⊕⊕⊕○ Moderate
Molar Buccal bone thickness, BBT_M1—immediate effect									
2	Randomised trials	Serious^a^, ^b^	Not serious	Not serious	Not serious	45	43	SMD −0.16 (*p*=0.467) (−0.57 to 0.26)	⊕⊕⊕○ Moderate
Short term Effect									
Sutural Expansion at Anterior Nasal Spine (ANS) region, SE_ANS—short term effect									
2	Randomised trials	Serious^a^	Very serious	Not serious	Not serious	39	37	SMD **1.12** **(*p*<0.001)** (0.61 to 0.63)	⊕○○○ Very low
Sutural Expansion at Poterior Nasal Spine (PNS) region, SE_PNS—short term effect									
3	Randomised trials	Serious^a^, ^d^	Serious	Not serious	Serious	69	68	SMD **0.27** (*p*=0.186) (−0.13 to 0.66)	⊕○○○ Very low
Nasal Width at premolar region, NW_PM1 – short term effect									
3	Randomised trials	Serious^a^, ^b^, ^d^	Not serious	Not serious	Not serious	60	58	SMD **0.97 (*p*=0.009)** (0.58 to 1.36)	⊕⊕⊕○ Moderate
Nasal Width at molar region, NW_M1—short term effect									
4	Randomised trials	Serious^a^, ^b^, ^d^	Not serious	Not serious	Not serious	72	71	SMD **1.18 (*p*<0.001)** (0.80 to 1.57)	⊕⊕⊕○ Moderate
Maxillary Width at premolar region, MW_PM1—short term effect									
3	Randomised trials	Serious^a^, ^b^, ^c^	Very serious	Not serious	Not serious	61	59	SMD **0.59 (*p*=0.005)** (0.18 to 1.01)	⊕○○○ Very low
Maxillary Width at molar region, MW_M1—short term effect									
4	Randomised trials	Very serious^a^, ^b^, ^c^, ^d^	Very serious	Not serious	Not serious	73	72	SMD **0.46 (*p*=0.010)** (0.11 to 0.82)	⊕○○○ Very low
Molar buccal tipping, TI_M1—short term effect									
4	Randomised trials	Serious^a^, ^b^, ^d^	Very serious	Not serious	Serious	72	71	SMD −0.22 (*p*=0.221) (−0.57 to 0.13)	⊕○○○ Very low
Premolar Buccal bone thickness, BBT_PM1—short term effect									
3	Randomised trials	Serious^a^, ^b^	Not serious	Not serious	Not serious	51	50	SMD **1.29 (*p*<0.001)** (0.86 to 1.73)	⊕⊕⊕○ Moderate
Molar Buccal bone thickness, BBT_M1—short term effect									
3	Randomised trials	Serious^a^, ^b^	Very serious	Not serious	Serious	51	50	SMD **0.68 (*p*=0.001)** (0.28 to 1.09)	⊕○○○ Very low
Long‐term Effect									
Sutural Expansion at Anterior Nasal Spine (ANS) region, SE_ANS—long‐term effect									
2	Randomised trials	Not serious	Not serious	Not serious	Serious	40	41	SMD −0.28 (*p*=0.209) (−0.72 to 0.16)	⊕⊕⊕○ Moderate
Sutural Expansion at Poterior Nasal Spine (PNS) region, SE_PNS—long‐term effect									
3	Randomised trials	Serious^c^	Not serious	Not serious	Serious	61	62	SMD −0.13 (*p*=0.481) (−0.48 to 0.23)	⊕⊕○○ Low
Nasal Width at premolar region, NW_PM1—long‐term effect									
1	Randomised trials	Not serious	Very serious	Not serious	Not serious	26	26	SMD **0.72 (*p*=0.012)** (0.16 to 1.29)	⊕⊕○○ Low
Nasal Width at molar region, NW_M1—long‐term effect									
2	Randomised trials	Serious^a^	Not serious	Not serious	Not serious	44	40	SMD **0.63 (*p*=0.005)** (0.19 to 1.08)	⊕⊕⊕○ Moderate
Maxillary Width at premolar region, MW_PM1—long‐term effect									
1	Randomised trials	Serious^b^, ^c^	Not serious	Not serious	very serious	21	21	SMD −0.51 (*p*=0.102) (−1.13 to 0.10)	⊕○○○ Very low
Maxillary Width at molar region, MW_M1—long‐term effect									
2	Randomised trials	Serious^a^, ^b^, ^c^	Serious	Not serious	Serious	39	35	SMD **0.21** (*p*=0.382) (−0.26 to 0.67)	⊕○○○ Very low
Molar buccal tipping, TI_M1—long‐term effect									
1	Randomised trials	Not serious	Very serious	Not serious	Not serious	26	26	SMD **−0.72 (*p*=0.012)** (−1.28 to −0.15)	⊕⊕○○ Low
Molar Buccal bone thickness, BBT_M1—long‐term effect									
1	Randomised trials	Not serious	Not serious	Not serious	Very serious	26	26	SMD **0** (*p*=1.000) (−0.54 to 0.54)	⊕⊕○○ Low

*Note:* Bold values are statistically significant.

Abbreviations: CI, confidence interval; SMD, standardised mean difference.

^a^ Serious concern in missing outcome data.

^b^ Serious concern in missing measurement of outcome.

^c^ Serious concern in randomization.

^d^ Serious concern in intended intervention.

## 4 Discussion

### 4.1 General Information

This systematic review has analysed the immediate, short‐term, and long‐term effects of bone‐anchored (BA) versus tooth‐borne (TB) expanders on the maxillary base and dento‐alveolar structures. A total of 358 patients from randomised clinical trials were included in the analysis. The GRADE assessment indicated the quality of evidence ranged from very low to moderate, primarily due to inadequate sample sizes, varying reference points, and significant potential risks of bias with inconsistent and imprecise results.

Data extraction for sutural expansion encountered challenges due to differing reference points in studies. For example, some studies measured changes at the nasopalatine foramen, which was deemed comparable to data from the anterior nasal spine region [9, 12, 21] due to their close anatomical proximity [52]. Similar approaches were taken for measurements at the posterior nasal spine region [52] (right and left lateral pterygoid plates [13, 20, 49], right and left greater palatine foramina [12, 21]).

Inevitably some outcome measurement has involved two measurements with the right and left side reported. The tooth inclination changes of the 1st premolars and 1st molars on the left and right side were measured [10, 13, 20, 22]. One study [22] measured the distance between cortical borders of the suture at the inferior and superior part. To obtain one value for meta‐analysis, the measured outcome was combined to one value respectively for further pooled analysis.

There are different papers using different reference points for dentoalveolar effects. Davami et al. [21] was excluded for buccal bone thickness measurement, as the buccal bone thickness at the root apex level was used instead of the cemento‐enamel junction level. Such value could be affected by the buccal tipping of molars after expansion and not comparable to the other data measured by the other studies [9, 12, 22, 49] that all measured at the cemento‐enamel junction or furcation level. Regarding the tooth inclination, some [9, 13] utilising the horizontal plane can be converted and be comparable to the other studies that use the sagittal plane for reference by using trigonometric calculations. However, Garib et al. [48] was excluded from pooled analysis, as the angle they used was between lines passing through the buccal and lingual molar cusp tips, at which it is not feasible to be converted for comparison.

Pasqua et al. [10] had employed ANCOVA (analysis of covariance) for intergroup comparison to adjust the data for the influence of the covariates on changes in expansion. Therefore, the original data about the changes in nasal width, maxillary width, and tooth inclination were utilised for meta‐analysis.

Only Jia et al. [13] assessed the mid‐palatal sutural opening and included those samples that failed to have the suture opened up. The other studies [12, 22, 48, 49] excluded samples that had lost follow‐up, screw instability or loss, or failure of sutural opening, potentially introducing bias.

### 4.2 Outcome of the Review and Its Agreement with Other Studies or Reviews

The present review revealed that BA expanders led to a greater amount of sutural expansion, consistent with Krusi et al. [27] findings. However, while BA expansion exhibited greater immediate nasal and maxillary width, Krusi et al. [27] found no significant differences. Furthermore, there was an overall greater increase in the nasal width and maxillary width at the premolar region, yet the result is inconsistent. Interestingly, the result has shown that there was no significant difference in the buccal bone thickness and 1st molar buccal tipping. This might probably be due to the separation of the two maxillary halves, which leads to alveolar bone bending. One trial by Jia et al. [13] has indicated that there is significantly less premolar buccal tipping, probably due to the appliance anchored on the 1st molars only.

For short‐term skeletal effects, BA expanders demonstrated a greater increase in nasal width compared to TB expanders [51, 53, 54] aligning with previous research [27], but not in maxillary width and sutural opening posteriorly. Inconsistencies were found regarding maxillary width changes at the premolar region [10, 12, 20], attributed to differences in expander design and expansion protocols (one with 4 turns on the first day of activation [10], with the others 2 turns per day) and whether using solely bone‐borne or hybrid ones.

In terms of short‐term dentoskeletal effects, the current review has indicated that the BA expander resulted in less reduction in the buccal bone thickness, consistent with Krusi et al.'s findings [27]. Although there is no statistically significant difference in the molar buccal tipping, there is significantly less buccal tipping in premolars, influenced by the appliance anchorage type and activation protocol. For example, Pasqua et al. [10] has indicated that there is more molar buccal tipping, as they performed one complete turn of expansion for the first activation. Further studies are needed to consolidate such factors. It appears that with time, the difference in treatment effects between the hybrid/bone‐borne and tooth‐borne expansion groups has become more insignificant, as indicated in the systematic review by Khosravi et al. [26].

There are insufficient studies for long‐term changes, with only a few studies showing no significant differences between groups over time. The overall enhancement of BA expanders tended to diminish, necessitating further research to confirm these findings. However, it is comprehensible that acquiring data on the long‐term dentoalveolar effects solely by expanders may be difficult, because it might not be appropriate for patients to delay bonding brackets for more than 6 months, which could otherwise influence the dentoalveolar effect.

The mean initial chronological age ranged widely from 9 to 15 years old, revealing heterogeneity. With age and progressive maturity, the increased interdigitation of the mid‐palatal suture might have caused the maxilla to be more resistant to expansion [55, 56], affecting the expansion result.

### 4.3 Quality of Evidence, Strength and Limitations

The strength of the present systematic review has included a comprehensive search strategy and tight selection criteria by including 10 randomised clinical trials that all involved a control group. Two reviewers have reviewed all articles and cross‐checked the data extraction. To the best of our knowledge, this is the first review that evaluated the immediate, short‐term, and long‐term effects after maxillary expansion. This has provided a better understanding of the difference in the treatment effect between the hybrid/bone‐borne and tooth‐borne expansion groups, at least for the immediate and short‐term effects.

There are limitations in this present review. Blinding of participants and operators was not possible as the use of the expander was apparent. Although only RCTs were included in this systematic review and meta‐analysis, there were five studies [12, 13, 20, 48, 49] out of ten having a high risk of bias, due to the handling of missing data and inconsistent reporting in the studies. Such a significant number of studies having potential risks of bias has affected the quality of the outcome be regarded from very low to moderate according to GRADE assessment. Thus, the results and conclusions of this systematic review should be considered with caution. Overall, the majority of the outcomes have revealed that the studies were characterised by significant heterogeneity, although *I* ^2^ and chi^2^ are subject to uncertainty and should be interpreted cautiously when the number of included studies is small. While more advanced measures (e.g., tau‐squared statistics or prediction intervals) may provide further context, we finally gave up adopting them due to the small number of included studies and the exploratory nature of this review: we aimed to “compare” the effects of the two appliances rather than obtaining a comprehensive absolute value of the effects. The significant heterogeneity could be due to the differences in the appliance design, activation protocol, age, sex, and demographic variability of patients for confounding variables. Subgroup analyses based on patients' age, different appliance designs, or activation protocols could be implemented to provide more detailed and accurate insights. However, the number of studies for subgroup analyses would be too limited for comparison and further analyses. For example, this meta‐analysis included adolescent patients across a range of ages, and conducting subgroup analyses or meta‐regression to investigate whether ages or other factors significantly influenced the results may provide important insights. However, as recommended in the Cochrane Handbook (section 10.11.5.1), when there are fewer than 10 studies included, such analyses should generally not be conducted as this risks generating unstable or misleading results. Therefore, we did not perform an analysis of effect modification by age, which should be expected to be done when there are more eligible studies in the future. In this study, we conducted influence analysis, which is a basic sensitivity analysis to assess the robustness of our findings. Compared with other sensitivity analyses, influence analysis can help evaluate how much an individual study affects the overall results, thereby helping us identify potentially influential studies that might skew the summary findings. Of note, we do acknowledge that with a small number of meta‐analysed studies, the interpretation of influence analysis must be approached with caution. In addition, some studies were using other reference points, which rendered the data not comparable for meta‐analysis. It was recommended that more future studies with fewer potential risks of bias can be conducted in a more standardised way, in terms of activation protocols, appliance designs, and retention periods to reduce the heterogeneity and enhance the reliability and clinical applicability of the results. The number of studies that can be included for meta‐analysis was insufficient, especially for the long term. There is a particular need for long‐term studies to investigate the changes in differences. Besides, the authors of the 10 selected studies did not analyse patient‐related outcome measures, such as overall patient satisfaction, pain or discomfort, or any other complications, which include the failure rate of the mini‐screws (except one study [13]) and appliance breakage. These details can not only facilitate a more comprehensive analysis of the overall impact of the two appliances but also help to understand the patients' acceptance of the appliances. In particular, the latter may influence patient compliance during treatment, thereby affecting the objective of the study results.

## 5 Conclusion

Current evidence suggests that hybrid/BA expanders produce significantly greater skeletal effect than tooth‐borne expanders in the immediate and short term. These effects include increased sutural opening and nasal width, alongside less premolar buccal tipping and greater buccal bone thickness at the premolar and molar regions less than 6 months after expansion. Over time, the differences between groups appear to diminish time, indicating potential instability in the long‐term outcomes. Due to the high heterogeneity among studies and low quality of evidence reported, these findings should be interpreted with caution. Further studies with more standardised methods would be needed, particularly for long‐term changes.

## Author Contributions

The first author (A.K.C.Y.) and second author (H.H.C.) performed study selection, data extraction, and risk of bias assessment independently and in duplicate. Literature search and data analysis were performed by the first author (A.K.C.Y.). Disagreements were resolved by discussion or involvement of the last author (Z.S.). J.L., K.F.H. and Z.T. revised the manuscript. All authors read and approved the final manuscript.

## Ethics Statement

Preferred Reporting Items for Systematic Reviews and Meta‐Analyses (PRISMA) statement and Cochrane Handbook for systematic reviews of interventions (http://ohg.cochrane.org).

## Conflicts of Interest

The authors declare no conflicts of interest.

## Supporting information


**Table S1:** Search Strategy.
**Table S2:** Excluded studies after retrieving the full text with reasons.
**Figure S1:** Forest plot for the immediate effect comparing the Hybrid or Bone‐borne and Tooth‐borne expansion, part a.
**Figure S2:** Forest plot for the immediate effect comparing the Hybrid or Bone‐borne and Tooth‐borne expansion, part b.
**Figure S3:** Forest plot for the immediate effect comparing the Hybrid or Bone‐borne and Tooth‐borne expansion, part c.
**Figure S4:** Forest plot for the short‐term effect comparing the Hybrid or Bone‐borne and Tooth‐borne expansion, part a.
**Figure S5:** Forest plot for the short‐term effect comparing the Hybrid or Bone‐borne and Tooth‐borne expansion, part b.
**Figure S6:** Forest plot for the short‐term effect comparing the Hybrid or Bone‐borne and Tooth‐borne expansion, part c.
**Figure S7:** Forest plot for the long‐term effect comparing the Hybrid or Bone‐borne and Tooth‐borne expansion.
**Figure S8:** Immediate effect, influence analysis.
**Figure S9:** Short term effect, influence analysis.
**Figure S10:** Long term effect, influence analysis.
**Figure S11:** Sensitivity test of the nasal width and maxillary width changes (Short‐term effect).

## Data Availability

The datasets used and/or analyzed during the current study are available from the corresponding author on reasonable request.
