# Supplementary material for: Comparative Three Dimensional Evaluation of Skeletal and Dento‐Alveolar Effects Between Tooth‐Borne and Bone‐Anchored Maxillary Expansion for Growing Patients—A Systematic Review and Meta‐Analysis
Source: Orthod Craniofac Res. 2025 Sep 27;28(6):907–28. doi: 10.1111/ocr.70029 (PMC12603678; doi:10.1111/ocr.70029)

**Supplementary Table S1**. Search strategy

| Database | Search strategy | Result |
| --- | --- | --- |
| PubMed | ((((((((((((((((palatal expansion technique[MeSH Terms]) OR (palatal expansion technique)) OR (maxillary expan*)) OR (skeletal expan*)) OR (palatal expan*)) OR (transverse expan*)) OR (transversal expan*)) OR (orthodontic expan*)) OR (transpalatal distract*)) OR (orthopaedic maxillary expan*)) OR (orthopedic maxillary expan*)) OR (rapid palatal disjunction)) OR (rapid maxillary disjunction)) OR (((RME) OR (rapid maxillary expan*)) OR (rapid palatal expan*)))))  AND  ((((((x-ray computed tomography[MeSH Terms]) OR (x-ray computed tomography)) OR ((((((((((((((((((((CAT scans) OR (CAT scan)) OR (CT scans)) OR (CT scan)) OR (computerized tomography)) OR (computerised tomography) ) OR (computed tomography)) OR (cone-beam computed tomography[MeSH Terms])) OR (computed tomography, volumetric[MeSH Terms])) OR (volumetric computed tomography)) OR (cb-ct)) OR (volumetric ct)) OR (cone beam)) OR (cone-beam)) OR (cone beam ct)) OR (cone-beam ct)) OR (three dimensional)) OR (digital volume tomography)) OR (digital volumetric tomography)) OR (volumetric tomography))) OR (volumetric radiography))))  AND  (((((adolescen*) OR (child*)) OR (grow*)) OR (teen*))) | 1142 |
| Embase | 1 palatal expansion/ 1212  2 palatal expansion technique.mp. 176  3 maxillary expan*.mp. 2321  4 skeletal expan*.mp. 182  5 palatal expan*.mp. 2025  6 transverse expan*.mp. 166  7 transversal expan*.mp. 42  8 orthodontic expan*.mp. 81  9 transpalatal distract*.mp. 58  10 orthopaedic maxillary expan*.mp. 2  11 orthopedic maxillary expan*.mp. 9  12 rapid palatal disjunction.mp. 2  13 rapid maxillary disjunction.mp. 0  14 RME.mp. 1875  15 rapid maxillary expan*.mp. 1574  16 rapid palatal expan*.mp. 772  17 1 or 2 or 3 or 4 or 5 or 6 or 7 or 8 or 9 or 10 or 11 or 12 or 13 or 14 or 15 or 16 4685  18 x-ray computed tomography.mp. 115273  19 CAT scans.mp. 331  20 CAT scan.mp. 1494  21 CT scans.mp. 75600  22 CT scan.mp. 142230  23 computerized tomography.mp. 31513  24 computerised tomography.mp. 3574  25 computed tomography.mp. 705172  26 computer assisted tomography/ or x-ray computed tomography/ 1074803  27 computer assisted tomography/ or cone beam computed tomography/ 999572  28 volumetric computed tomography.mp. 397  29 cb-ct.mp. 141  30 volumetric ct.mp. 984  31 cone beam.mp. 37009  32 cone-beam.mp. 37009  33 cone beam ct.mp. 9331  34 cone-beam ct.mp. 9331  35 three dimensional.mp. 371169  36 digital volume tomography.mp. 145  37 digital volumetric tomography.mp. 9  38 volumetric tomography.mp. 85  39 volumetric radiography.mp. 1  40 18 or 19 or 20 or 21 or 22 or 23 or 24 or 25 or 26 or 27 or 28 or 29 or 30 or 31 or 32 or 33 or 34 or 35 or 36 or 37 or 38 or 39 1697470  41 adolescen*.mp. 2152576  42 child*.mp. 3695001  43 grow*.mp. 3780003  44 teen*.mp. 54104  45 41 or 42 or 43 or 44 8102720  46 17 and 40 and 45 617 | 617 |
| Medline | 1 palatal expansion/ 0  2 palatal expansion technique.mp. 3463  3 maxillary expan*.mp. 2202  4 skeletal expan*.mp. 180  5 palatal expan*.mp. 3724  6 transverse expan*.mp. 163  7 transversal expan*.mp. 37  8 orthodontic expan*.mp. 79  9 transpalatal distract*.mp. 46  10 orthopaedic maxillary expan*.mp. 3  11 orthopedic maxillary expan*.mp. 13  12 rapid palatal disjunction.mp. 2  13 rapid maxillary disjunction.mp. 1  14 RME.mp. 1754  15 rapid maxillary expan*.mp. 1511  16 rapid palatal expan*.mp. 730  17 1 or 2 or 3 or 4 or 5 or 6 or 7 or 8 or 9 or 10 or 11 or 12 or 13 or 14 or 15 or 16 5383  18 x-ray computed tomography.mp. 5224  19 CAT scans.mp. 233  20 CAT scan.mp. 914  21 CT scans.mp. 46580  22 CT scan.mp. 72665  23 computerized tomography.mp. 24035  24 computerised tomography.mp. 2315  25 computed tomography.mp. 387871  26 computer assisted tomography/ or x-ray computed tomography/ 430048  27 computer assisted tomography/ or cone beam computed tomography/ 15408  28 volumetric computed tomography.mp. 299  29 cb-ct.mp. 90  30 volumetric ct.mp. 629  31 cone beam.mp. 24859  32 cone-beam.mp. 24859  33 cone beam ct.mp. 5497  34 cone-beam ct.mp. 5497  35 three dimensional.mp. 313526  36 digital volume tomography.mp. 111  37 digital volumetric tomography.mp. 6  38 volumetric tomography.mp. 75  39 volumetric radiography.mp. 1  40 18 or 19 or 20 or 21 or 22 or 23 or 24 or 25 or 26 or 27 or 28 or 29 or 30 or 31 or 32 or 33 or 34 or 35 or 36 or 37 or 38 or 39 1000478  41 adolescen*.mp. 2378258  42 child*.mp. 2878892  43 grow*.mp. 2874480  44 teen*.mp. 37978  45 41 or 42 or 43 or 44 6737064  46 17 and 40 and 45 581 | 581 |
| Cochrane Central Register of Controlled Trials | #1 MeSH descriptor: [Palatal Expansion Technique] explode all trees 346  #2 palatal expansion technique 373  #3 maxillary expan* 679  #4 skeletal expan* 595  #5 palatal expan* 499  #6 transverse expan* 421  #7 transversal expan* 59  #8 orthodontic expan* 415  #9 transpalatal distract* 2  #10 orthopaedic maxillary expan* 67  #11 orthopedic maxillary expan* 67  #12 rapid palatal disjunction 12  #13 rapid maxillary disjunction 13  #14 RME 320  #15 rapid maxillary expan* 439  #16 rapid palatal expan* 354  #17 #1 or #2 or #3 or #4 or #5 or #6 or #7 or #8 or #9 or #10 or #11 or #12 or #13 or #14 or #15 or #16 1471  #18 MeSH descriptor: [Tomography, X-Ray Computed] explode all trees 8849  #19 x-ray computed tomography 9527  #20 CAT scans 83  #21 CAT scan 157  #22 CT scans 5876  #23 CT scan 10787  #24 computerized tomography 1628  #25 computerised tomography 1628  #26 computed tomography 24520  #27 MeSH descriptor: [Cone-Beam Computed Tomography] explode all trees 669  #28 volumetric computed tomography 711  #29 cb-ct 12  #30 volumetric ct 856  #31 cone beam 2146  #32 cone-beam 2117  #33 cone beam ct 772  #34 cone-beam ct 765  #35 three dimensional 8050  #36 digital volume tomography 746  #37 digital volumetric tomography 44  #38 volumetric tomography 920  #39 volumetric radiography 80  #40 #18 or #19 or #20 or #21 or #22 or #23 or #24 or #25 or #26 or #27 or #28 or #29 or #30 or #31 or #32 or #33 or #34 or #35 or #36 or #37 or #38 #39 41981  #41 adolescen* 181395  #42 child* 234518  #43 grow* 78232  #44 teen* 4281  #45 #41 or #42 or #43 or #44 403638  #46 #17 and #40 and #45 217 | 217 |
| Web of Science (WOS) | 1: ((((((((((((((ALL=(palatal expansion technique)) OR ALL=(maxillary expan*)) OR ALL=(skeletal expan*)) OR ALL=(palatal expan*)) OR ALL=(transverse expan*)) OR ALL=(transversal expan*)) OR ALL=(orthodontic expan*)) OR ALL=(transpalatal distract*)) OR ALL=(orthopaedic maxillary expan*)) OR ALL=(orthopedic maxillary expan*)) OR ALL=(rapid palatal disjunction)) OR ALL=(rapid maxillary disjunction)) OR ALL=(RME)) OR ALL=(rapid maxillary expan*)) OR ALL=(rapid palatal expan*)  2: ALL=(x-ray computed tomography) OR ALL=(CAT scan) OR ALL=(CT scan) OR ALL=(computerized tomography) OR ALL=(computerised tomography) OR ALL=(computed tomography) OR ALL=(cone-beam computed tomography) OR ALL=(computed tomography, volumetric) OR ALL=(volumetric computed tomography) OR ALL=(cb-ct) OR ALL=(volumetric ct) OR ALL=(cone beam) OR ALL=(cone-beam) OR ALL=(three dimensional) OR ALL=(digital volume tomography) OR ALL=(digital volumetric tomography) OR ALL=(volumetric tomography) OR ALL=(volumetric radiography)  3: (((ALL=(adolescen*)) OR ALL=(child*)) OR ALL=(grow*)) OR ALL=(teen*)  4: #1 AND #2 AND #3 | 823 |

Supplementary Table S2 Excluded studies after retrieving the full text with reasons

| No control group or the comparison group is not tooth-borne expander, n=9 | Kim K.A. et al 2019 |
| --- | --- |
|  | Annarumma F et al 2021 |
|  | Buyuk S.K. et al 2022 |
|  | Jia H. et al 2022 |
|  | Kapetanovic A. et al 2022 |
|  | Liao Y.C. et al 2022 |
|  | McMullen C. et al 2022 |
|  | Mendoza P.S. et al 2022 |
|  | Yacout Y.M. et al 2022 |
| Not Randomized or non-randomized clinical trials, n=15 | Farhangfar A. et al 2012 |
|  | Lin L. et al 2015 |
|  | Mosleh M.I. et al 2015 |
|  | Lemos Rinaldi M.R. et al 2018 |
|  | Lo Giudice et al 2020 |
|  | Moon H.W. et al 2020 |
|  | Cremonini F et al 2021 |
|  | Altieri F et al 2022 |
|  | Nallamilli L. et al 2022 |
|  | Ronsivalle V. et al 2022 |
|  | Venezia et al 2022 |
|  | Karaman A. et al 2023 |
|  | Ning R. et al 2023 |
|  | Nie X. et al 2023 |
|  | Yi F. et al 2023 |
| Combined studies or same samples, n=2 | Lagravere et al 2013 |
|  | Lo Guidice et al 2020 |
| No CBCT evaluation, n=3 | Yilmaz A. et al 2015 |
|  | Malmvind D. et al 2022 |
|  | Abdelsalam R. et al 2023 |

Supplementary Figure SF1 - Forest plot for the immediate effect T1-T0 comparing Hybrid or Bone-borne (BB) and Tooth-borne expansion (TBB), part a


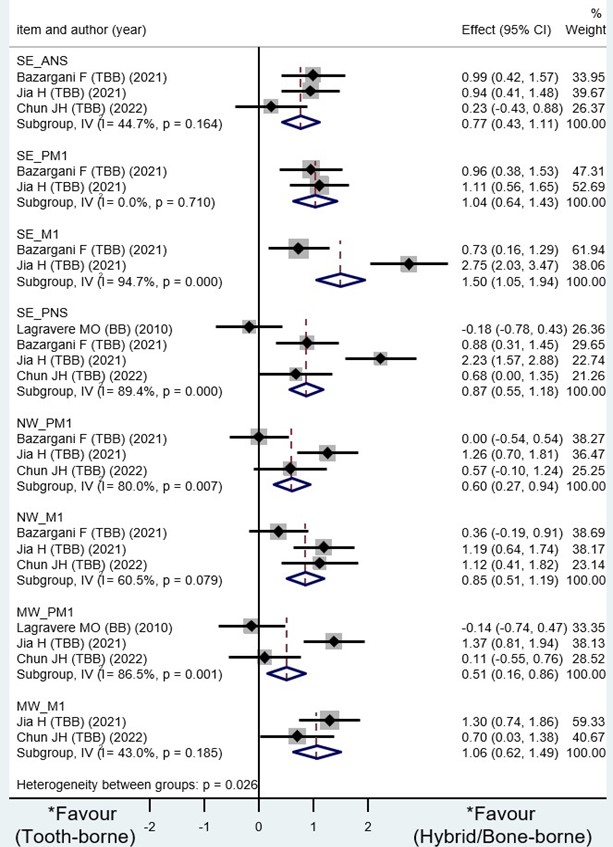


Supplementary Figure SF2 - Forest plot for the immediate effect T1-T0 comparing the Hybrid or Bone-borne (BB) and Tooth-borne expansion (TBB), part b


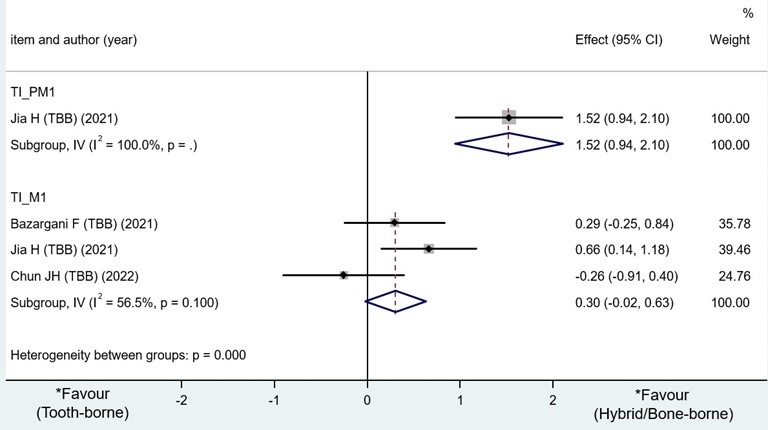


Supplementary Figure SF3 - Forest plot for the immediate effect T1-T0 comparing the Hybrid or Bone-borne (BB) and Tooth-borne expansion (TBB), part c


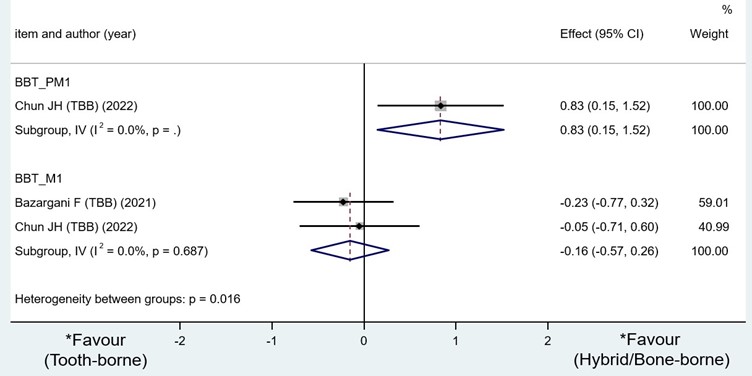


Supplementary Figure SF4 - Forest plot for the short-term effect T2-T0 comparing the Hybrid or Bone-borne (BB) and Tooth-borne expansion (TBB), part a


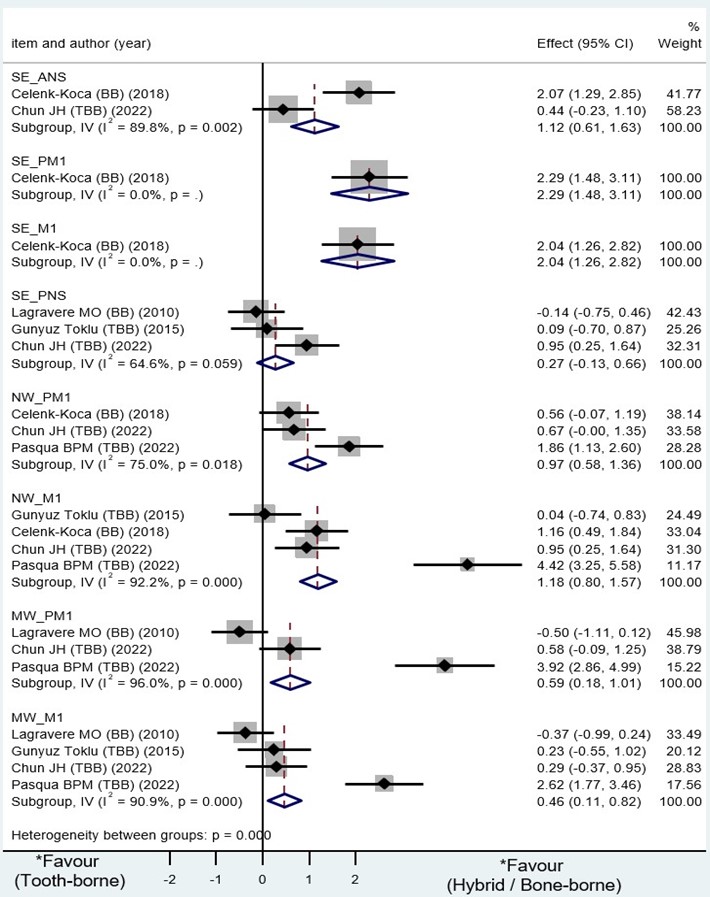


Supplementary Figure SF5 - Forest plot for the short-term effect T2-T0 comparing the Hybrid or Bone-borne (BB) and Tooth-borne expansion (TBB), part b


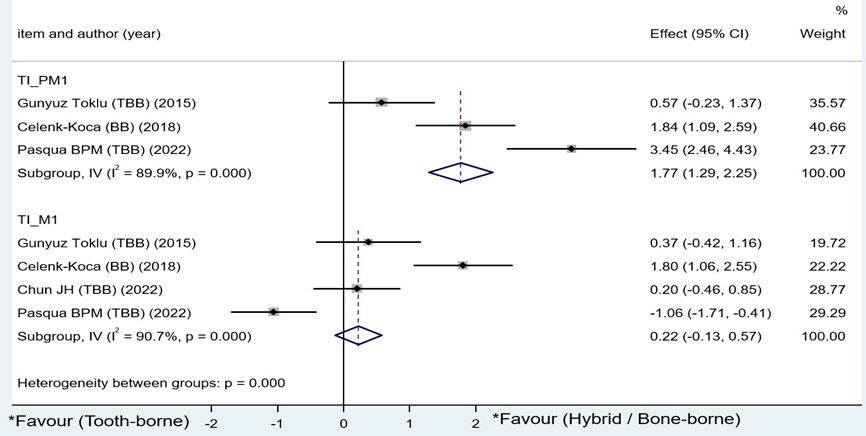


Supplementary Figure SF6 - Forest plot for the short-term effect T2-T0 comparing the Hybrid or Bone-borne (BB) and Tooth-borne expansion (TBB), part c


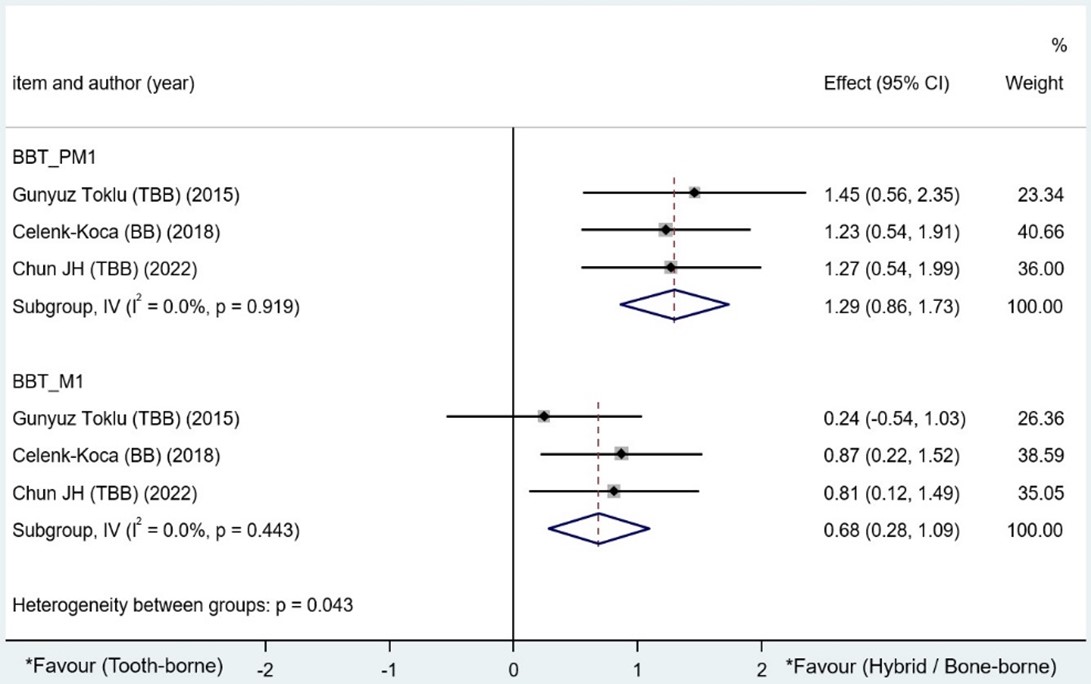


Supplementary Figure SF7 - Forest plot for the long-term effect T3-T0 comparing the Hybrid or Bone-borne (BB) and Tooth-borne expansion (TBB)


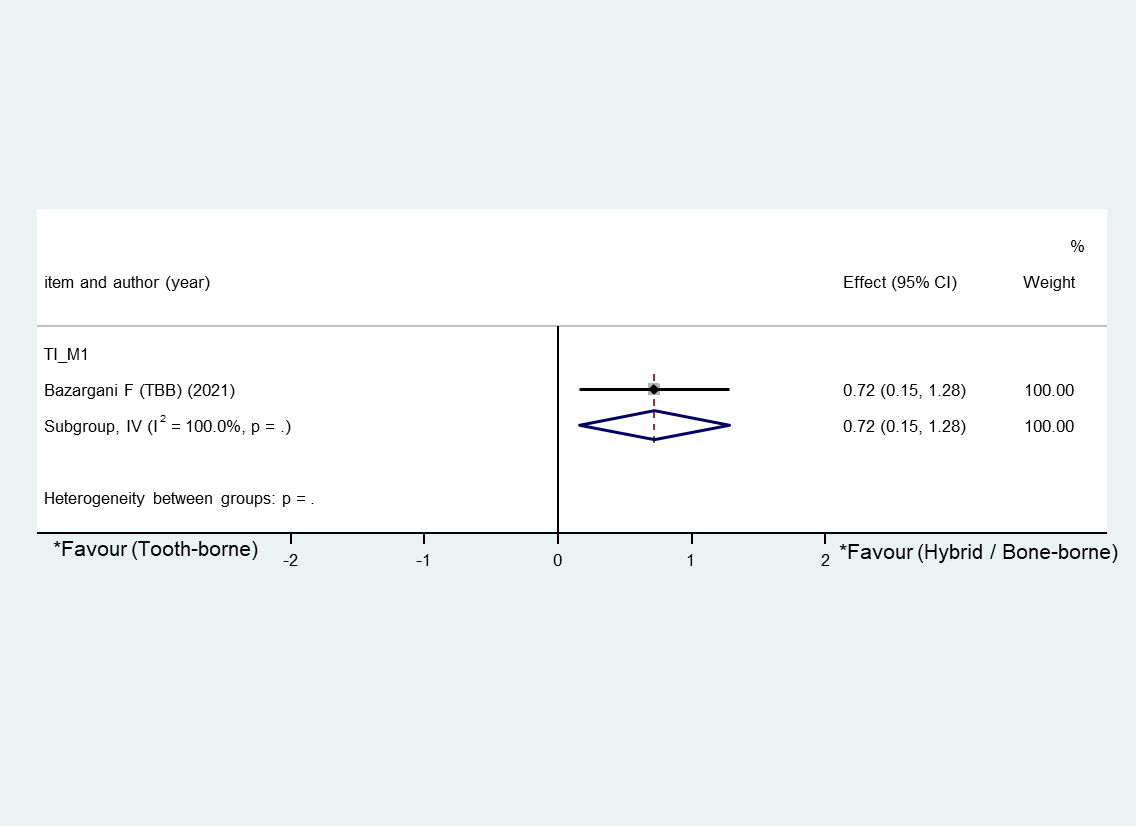

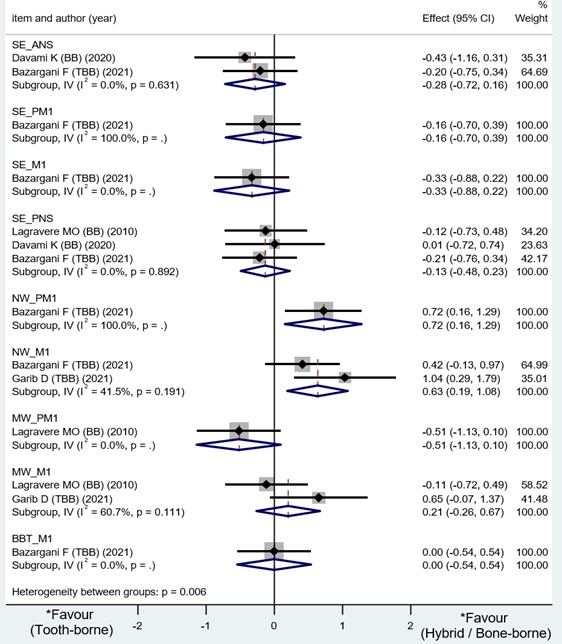


Supplementary figure SF8 (immediate effect, influence analysis) comparing Hybrid or Bone-borne (BB) and Tooth-borne expansion (TBB)


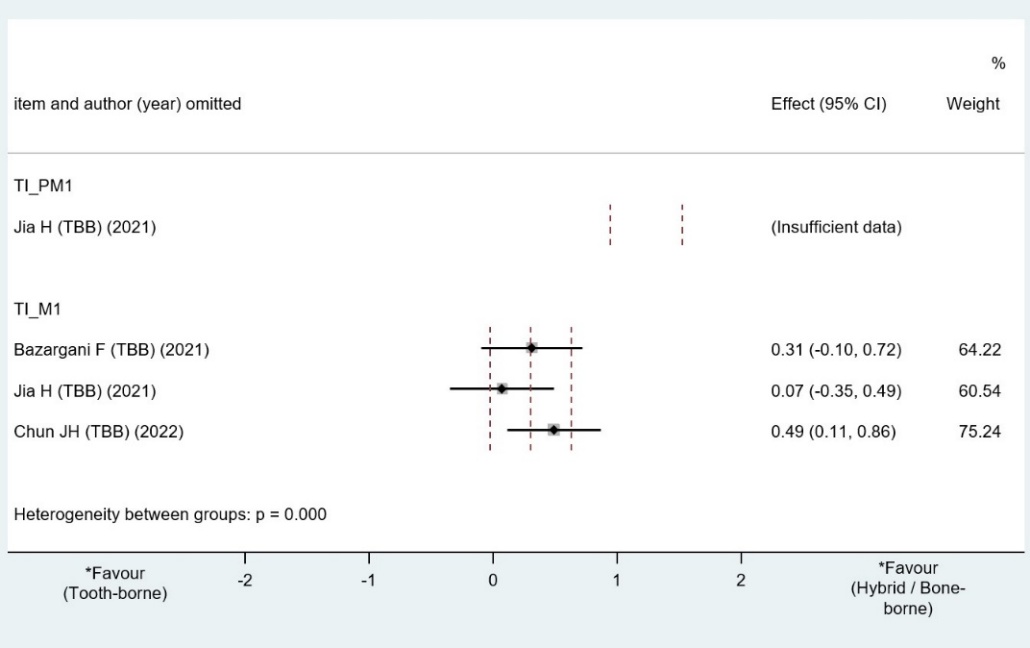

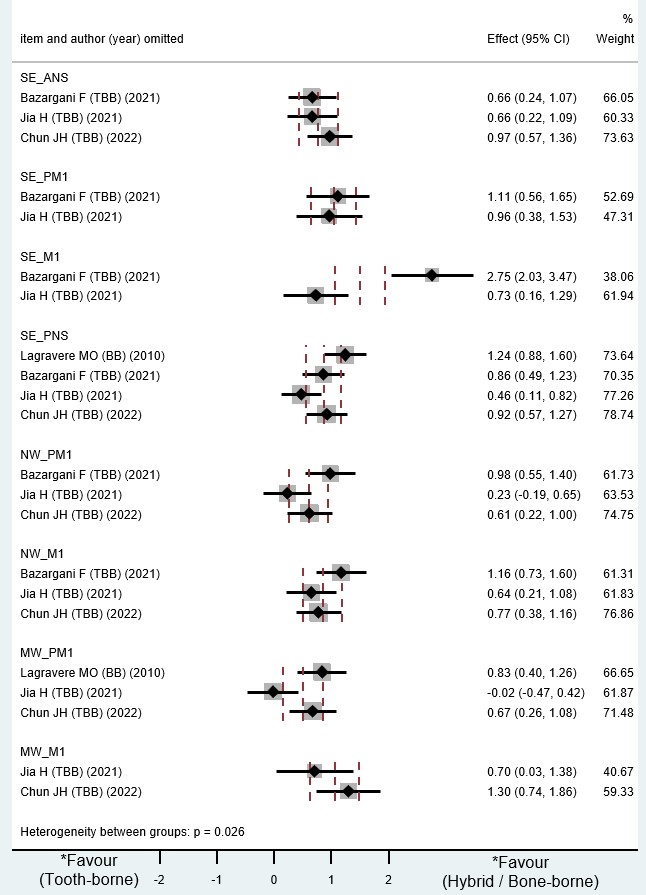
Supplementary figure SF9 (short term effect, influence analysis) comparing Hybrid or Bone-borne (BB) and Tooth-borne expansion (TBB)


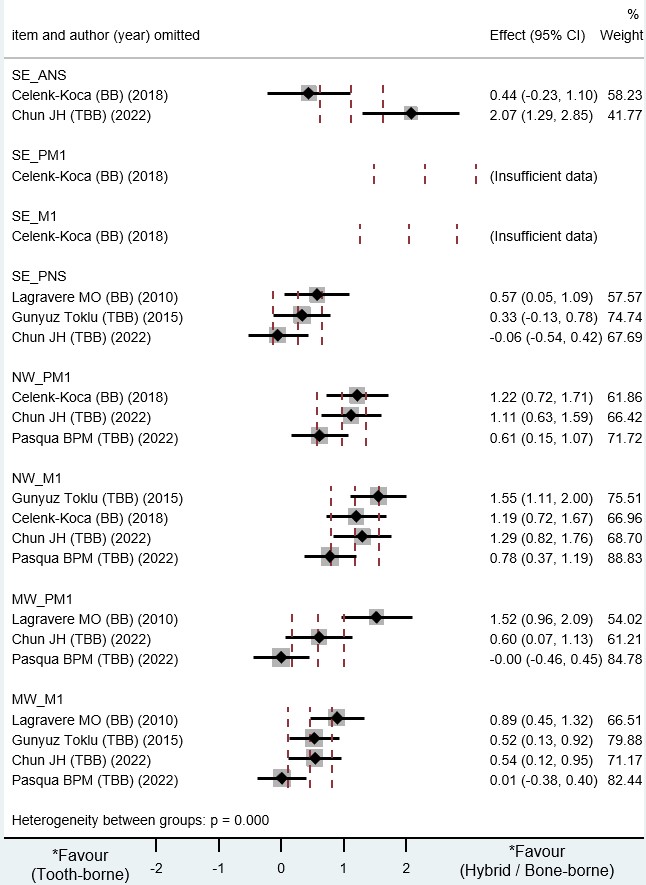


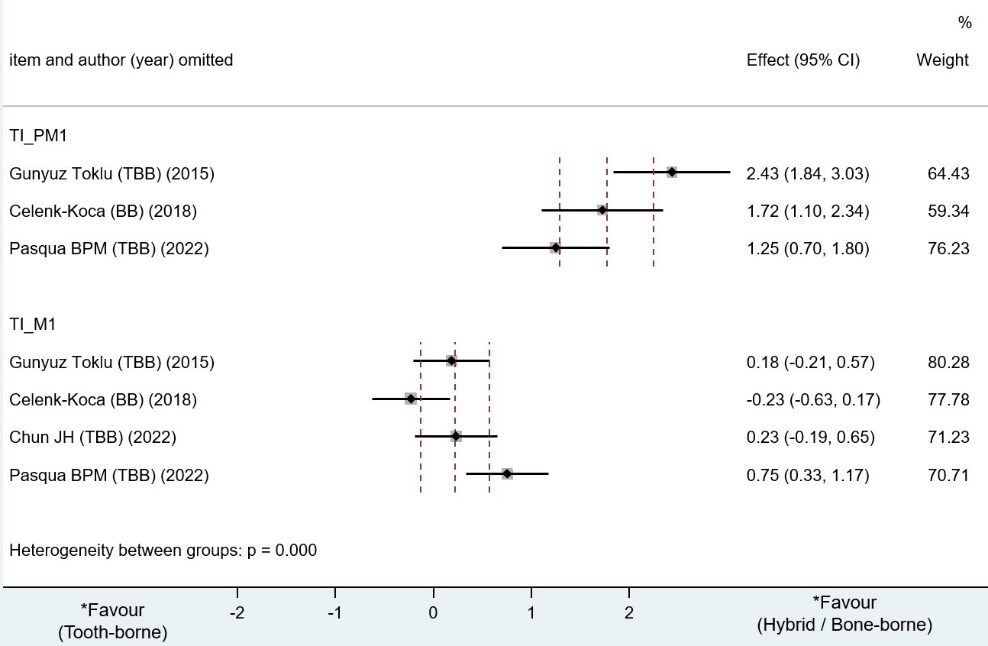


Supplementary figure SF10 (long term effect, influence analysis) comparing Hybrid or Bone-borne (BB) and Tooth-borne expansion (TBB)


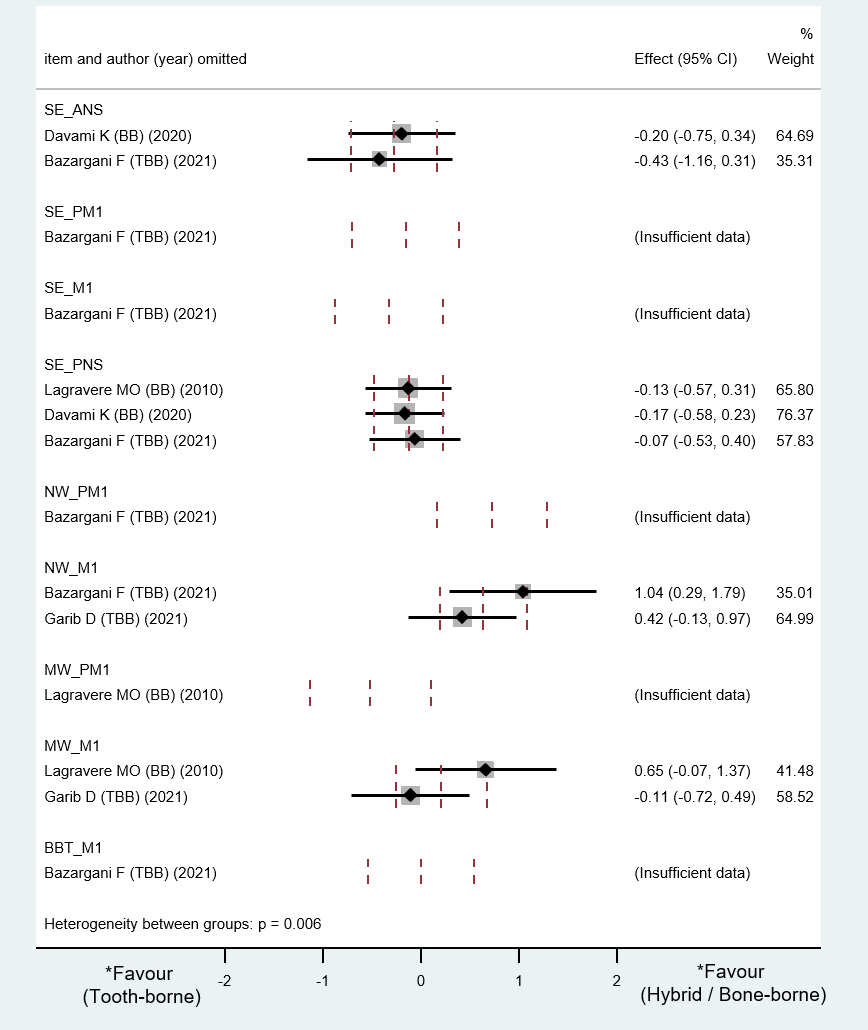


Supplementary Figure SF11 - Sensitivity test of the nasal width and maxillary width changes (Short-term effect) comparing Hybrid or Bone-borne (BB) and Tooth-borne expansion (TBB)


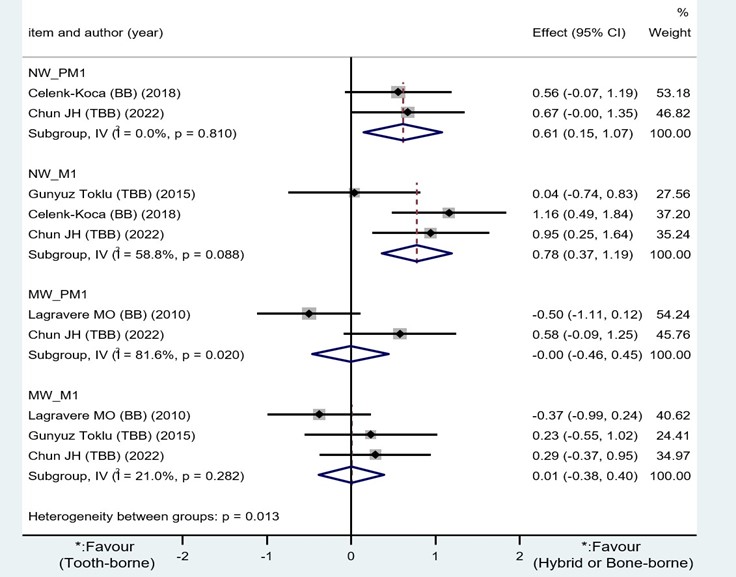

Supplement: Supplementary file 1 — Table S1: Search Strategy. Table S2: Excluded studies after retrieving the full text with reasons. Figure S1: Forest plot for the immediate effect comparing the Hybrid or Bone‐borne and Tooth‐borne expansion, part a. Figure S2: Forest plot for the immediate effect comparing the Hybrid or Bone‐borne and Tooth‐borne expansion, part b. Figure S3: Forest plot for the immediate effect comparing the Hybrid or Bone‐borne and Tooth‐borne expansion, part c. Figure S4: Forest plot for the short‐term effect comparing the Hybrid or Bone‐borne and Tooth‐borne expansion, part a. Figure S5: Forest plot for the short‐term effect comparing the Hybrid or Bone‐borne and Tooth‐borne expansion, part b. Figure S6: Forest plot for the short‐term effect comparing the Hybrid or Bone‐borne and Tooth‐borne expansion, part c. Figure S7: Forest plot for the long‐term effect comparing the Hybrid or Bone‐borne and Tooth‐borne expansion. Figure S8: Immediate effect, influence analysis. Figure S9: Short term effect, influence analysis. Figure S10: Long term effect, influence analysis. Figure S11: Sensitivity test of the nasal width and maxillary width changes (Short‐term effect). [file OCR-28-907-s001.docx]
